# Zinc oxide seed priming enhances drought tolerance in wheat seedlings by improving antioxidant activity and osmoprotection

**DOI:** 10.1038/s41598-025-86824-z

**Published:** 2025-01-31

**Authors:** Rasha M. El-Shazoly, A. A. Othman, Muhammad Saqlain Zaheer, Ahmed F. Al-Hossainy, Dalia A. Abdel-Wahab

**Affiliations:** 1 Botany and Microbiology Department, Faculty of Science, New Valley University, Al-Kharja, New Valley 72511 Egypt; 2 Physics Department, Faculty of Science, Assiut University, Assiut, 71515 Egypt; 3 Department of Agricultural Engineering, Khwaja Fareed University of Engineering and Information Technology, Rahim Yar Khan, Pakistan; 4 Chemistry Department, Faculty of Science, New Valley University, Al-Wadi Al-Gadid, Al-Kharga, 72511 Egypt; 5 Chemistry Department, Faculty of Science, Northern Border University, 1321 Arar, Saudi Arabia

**Keywords:** Nanopriming, ZnO NPs, ZnO bulk, Drought stress, Antioxidant system, Free radical scavenging abilities, *Triticum aestivum*, TD-DFT calculations, Physiology, Plant sciences, Climate sciences, Nanoscience and technology

## Abstract

Drought can affect all growth stages and has a significant effect on seed germination, which affects all physiological and metabolic germination processes. It also leads to dehydration, which increases the oxidation of lipids and membranes and disrupts the functioning of biomolecules in plants. Zinc is an essential element for several enzymes involved in metabolism, cell elongation, preservation of the strength and integrity of cell membranes, seed development, and resistance to environmental stress. A pot experiment was conducted to determine how ZnO seed priming, either in the form of ZnO NPs (nanopriming) or ZnO bulk priming (60 mg L^− 1^), counteracts the negative impacts of drought at different levels (80% and 60% FC) on wheat (*Triticum aestivum* L.) seedlings at the seedling stage. A recent experiment revealed that seed priming agents significantly mitigate the negative effects of drought stress, especially at 60% FC, by positively influencing various parameters of wheat seedlings. Notably, the POD activity increased by 91.8% and 289.9% for the shoots, 218.6% and 261.6% for the roots, the phenolic content increased by 194.4% for the shoots and 1139.6% for the roots, the H_2_O_2_ scavenging percentage increased by 124.9% and 135.4% for the shoots and 147.6% for the roots, and the lipid peroxidation inhibition percentage increased by 320.6% and 433% for the shoots. Moreover, the utilization of seed priming agents had a profound effect on free amino acids (393.8%, 502.8% for roots) and soluble carbohydrates (183.4% for roots) compared with those in stressed seedlings without priming. Experimental and computational methods (time-dependent density functional theory (TD-DFT)) were employed to perform IR and XRD analyses of the isolated molecules of the ZnO NPs/Iso. In conclusion, the application of ZnO NPs or bulk ZnO was found to create effective mechanical and physiological barriers, as confirmed by the analysis of antioxidant enzyme activities, nonenzymatic components, free radical scavenging, and osmoprotectant constituents.

## Introduction

Drought stress affects agricultural productivity and is one of the most severe abiotic stresses. The primary cause of the frequent emergence of drought stress worldwide is the unpredictability of the global climate with variable rainfall patterns^1^. Although inadequate precipitation is typically the main cause of drought stress, the evaporation of soil water may aggravate an already severe drought stress event because of high temperatures, dry winds, and strong sunshine^2^. Egypt has a total land area of 100 million ha (Mha), with deserts covering approximately 96% of it. Furthermore, the total cultivated area is 3.78 Mha. This area includes 2.25 Mha of old land and 1.53 Mha of newly reclaimed land that lack nutrients and water irrigation. Additionally, it is commonly recognized that the quantity of organic matter in soil declines in arid climatic zones^3^.

Although drought can affect all growth stages, it also significantly impacts seed germination^4,5^. Both physiological and metabolic germination processes are affected when the amount of water that flows into the seed decreases due to hydraulic reduction^6^. Because of inadequate water transfer from the xylem to the cells in conditions of severe shortages of water, cell division, elongation, and expansion are restricted, which decreases plant height, leaf area, stem extension, and root proliferation^7^. When plants are subjected to drought stress, they release reactive oxygen species (ROS), which cause various types of cell damage, including DNA fragmentation, lipid peroxidation, protein degradation, and the disruption of photosystems^8^. Moreover, drought stress results in increased production of reactive oxygen species (ROS), particularly in chloroplasts. These ROS include superoxide (O^2−^), hydroxyl (OH^−^), hydrogen peroxide (H_2_O_2_), singlet oxygen (O_2_^1−^), and alkoxy radical (RO)^9,10^.

Wheat (*Triticum aestivum* L.) is a good source of nutrients, particularly in regions with limited resources^11^. It supplies more than 40% of the global food demand and has high protein and carbohydrate contents^12^. A lack of water has affected the yield of wheat by approximately 65 million hectares throughout the last decade^13^. Wheat may grow in semiarid and arid areas; thus, irrigation management is necessary^14^. The storage of water in the case of wheat at the seedling stage, the stress affecting the plant during vegetative growth, and lethal stress are the key dangers. At any stage of growth, plants may encounter drought stress. The most prominent sensitive stages for crop development affected by abiotic stresses are the sprouting of seeds and the establishment of seedlings^15^. Thus, increasing the resistance of wheat to stress and identifying the mechanisms underlying the response of plants to drought are needed to increase yield^16^.

Zinc (Zn) plays a crucial role in plant development, influencing various growth aspects positively and negatively. It is involved in vital biochemical processes such as protein synthesis, chlorophyll production, and enzyme activity^17^. Although different NPs tend to use atypical techniques to alleviate the detrimental effects of drought stress, there are research and knowledge gaps concerning the best source and dose of NPs used as SP (seed priming) agents. There is currently insufficient knowledge regarding how nanopriming influences wheat plant growth, early stand development, and seed germination under both ordinary and stressed conditions^18^. Crops could benefit from the advantages of nanofertilizers or nanoencapsulated nutrients, which govern the release of chemical fertilizers that control plant growth and increase the desired activity^19^. Seed priming is an exogenous application of mineral nutrients, growth regulators, and osmoprotectants to mitigate the adverse effects of stress. There are many types of priming techniques and their mechanisms: hydropriming, osmopriming, chemopriming, and redox priming^20–22^. A previous study reported several advantages of priming, including consistency, early emergence, quick germination through a wide range of temperatures, the establishment of crops, and effective water usage. Primed seeds are also more economical and encourage deeper root growth^23^. The use of nanomaterials, mainly NPs, as priming agents in nanopriming has been recognized as a recent seed treatment technique^24^. Seed priming techniques, such as NP priming, have proven effective owing to their discrete physiochemical features and small size^25,26^. Because of their greater surface area, NPs can create new holes or enlarge existing roots, which enhances the movement of nutrients and water throughout plants and promotes their growth and development^27^. Since early drought is one of the greatest challenges for wheat cultivation in semiarid regions, it results in weak seedlings and poor growth. In extreme cases, drought may lead to crop failure with a reduced final yield. Therefore, maintaining resilience under conditions of declining water supply is an extremely challenging task^15^. In recent years, the trend toward the use of alternatives to organic fertilizers has shifted to the use of many mineral and nano fertilizers because of their minimal or nonexistent harm to soil. Compared with that of bulk-scale ZnO, the impact of nanoscale ZnO is greater in wheat under drought stress^28^. In contrast, ionic and nanoscale zinc contents do not significantly vary in drought-stressed soybeans, with the exception of increased zinc accumulation caused by ionic zinc treatment^29^. According to Wang et al.^30^, one of the main outcomes of nanoscale ZnO in plant–soil systems is its quick disintegration into ions. The amount of organic matter in the soil, however, can affect how easily plants absorb nanoscale ZnO.

In support of experimental results and to delve deeper into the mechanism and interaction underlying the adsorption process, theoretical investigations are frequently conducted. To the best of the researcher’s knowledge, no prior research has investigated the utilization of the oxidation process and incorporated theoretical approaches (DMOl^3^/TD-DFT) to optimize parameters and comprehend the oxidation process in an aqueous environment^31–33^. To optimize the oxidation process in an aqueous system through the application of a statistical method that depends on theoretical approaches and (DMOl^3^/TD-DFT) calculations, the objective of this study was to analyze and discuss the process by which the oxidation of SCM to oxalic acid and glyoxylic acid utilizes both spectroscopy and theoretical approaches^34–36^. To our knowledge, little research has been published on seed priming with nanoparticles and their role in stress management as a prominent strategy for climate change scenarios. In this study, two accessible ZnO sources, bulk and nano, were compared for their ability to stimulate the physiological and biochemical processes of wheat plants under drought conditions.

We evaluated the efficiency of priming seeds with ZnO NPs or ZnO bulk in drought adaptation by (1) growth and biomass yield; (2) photosynthetic pigment concentrations; (3) the formation of reactive oxygen species (ROS) and inhibition of membrane lipid peroxidation%; (4) antioxidant enzyme activities; (5) antioxidant capacities and nonenzymatic components; and (6) the build-up of osmoprotectants in wheat seedlings. TD-DFT calculations were employed to theoretically examine the fabrication of ZnO NPs to provide additional support for the experimental findings. The TD-DFT method was utilized to determine the chemical properties of the resulting complex.

## Materials and methods

### Source and characterization of ZnO nanoparticles and suspension preparation

**Fig. 1:**
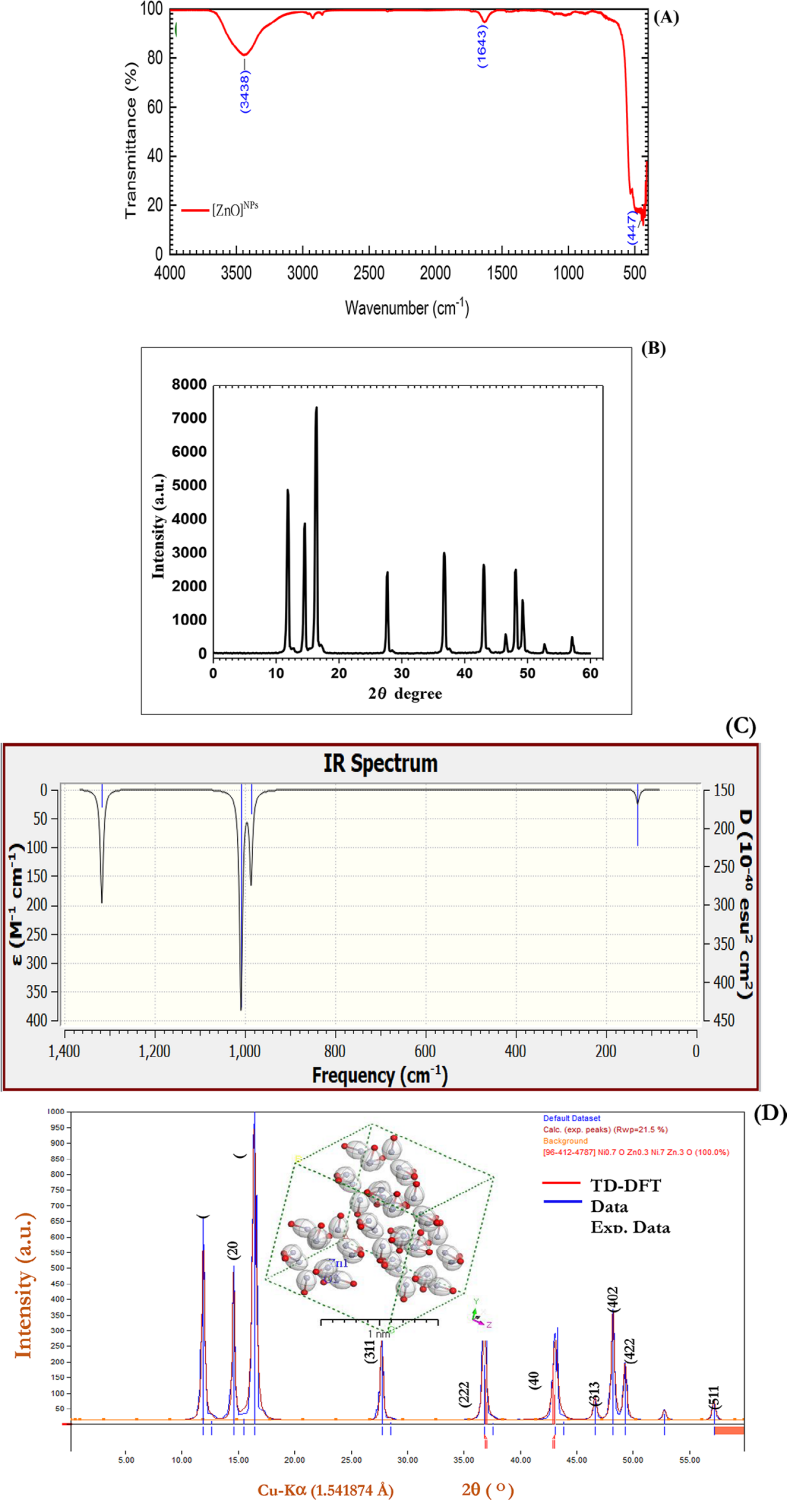
The experimental part for FTIR spectra and structure of ZnO NPs (**A**), XRD patterns of ZnO nanocrystals (**B**). Simulation IR by utilizing Gaussian 09 W Software with DFT/WB97XD and basis set 6–311 G for ZnO NPs/Iso Figure (inset) is a 3D Orthorhombic lattice-type and 3D Cubic lattice-type/F23(169) for ZnO NPs/Iso. (**C**). Combined between the experimental ZnO NPs and simulated XRD patterns ZnO NPs/Iso. Figure (inset) is a 3D Orthorhombic lattice-type and 3D cubic lattice-type F23 (196) for the simulated nanoparticles (**D**).

In the present work, ZnO nanoparticles with a size of 37 ± 2.6 nm were prepared *via* ball-milling techniques via commercial ZnO purchased from Chem Lab nv Company, Belgium (99.5% purity, 81.37 g/mol and a density of 5.61 g/cm^3^). A previous study Othman et al.^37^ used a PW 1700 X-ray diffractometer (XRD) with Cu Kα radiation (λ = 0.154056 nm) at a diffraction angle (2θ) in the range of 20–70° with a step size of 0.06 and refined it with crystal structure Celref software (Fig. 1A). Additionally, in a previous study, the surface morphology of purchased and milled ZnO NPs was examined by scanning electron microscopy (SEM) (Shimadzu Superscan SSX-550). Measurements of optical absorption and transmittance spectra were carried out *via* a UV–vis Perkin-Elmer Lambda 750 spectrophotometer in the range of 200–700 nm and Fourier transform infrared spectroscopy (Nicolet IS 10 FT-IR) (Fig. 1B). The tested ZnO nano- and ZnO bulk solutions were used at a concentration of 60 mg/L and employed as priming agents, after which they were sonicated for 30 min to ensure dispersion in the solution.

### Computational chemistry of the ZnO NPs *via* TD-DFT/DMol^3^ methods

Time-dependent density functional theory (TD-DFT) is an effective theoretical tool for investigating the fabrication process of zinc oxide nanoparticles. The three-dimensional (3D) structures of the ZnO NPs/Iso in an isolated state were obtained from the Chem/3D software program. The ZnO NPs/Iso potential energy curve (PES) was computed *via* Materials Studio 7.0 and the TD-DFT/DMol3 method. All the studied 3D structures of the ZnO NPs/Iso were optimized via the software materials Studio 7.0 program with DFT B3LYP 6-311 + + G (d, p). The B3LYP method is a hybrid functional method that was developed by combining Beckee’s gradient function and the Hartree − Fock local exchange function to calculate the energy of intermolecular hydrogen bonding interactions (E_Int/H_)^38^.

### Plant material and priming treatments

The grains of wheat (*Triticum aestivum* L.) of the Giza 168 cultivar were kindly supplied by the Agricultural Research Center, Giza, Egypt. The grains were surface sterilized in 10% hydrogen peroxide (H_2_O_2_) for 15 min, rinsed thoroughly with distilled water, and then soaked in priming agents (ZnO NPs 60 mg L^− 1^ or ZnO bulk 60 mg L^− 1^) for 12 h (according to previous preliminary experiments demonstrating that this time is an effective suitable priming time). After that, the primed seeds were left for 1 h on filter paper for dehydration in the air at room temperature. Then, 10 grains were sown at separate locations per pot at depths of approximately 0.5 cm. Plastic pots lined with polyethylene bags were filled with 0.5 kg of air-dried soil composed of mixed sieved air-dried clay and sand (1:2 by volume). The physical and chemical properties of the experimental soil are shown in Table 1.

**Table 1:** Physical and chemical properties of the experimental soil.

Sand	65%
Silt	17%
Clay	18%
Soil texture	Sandy loam
Organic matter	9.3 g kg^− 1^
PH	7.4
EC	2.97 ds m^− 1^
Cl^−^	0.446 mg g^− 1^
Na^+^	70 mg g^− 1^
K^+^	23 mg g^− 1^
Ca^+ 2^	0.165 mg g^− 1^

### Growth conditions and treatments

The grains were cultivated in a greenhouse under natural growing conditions. Table 2 shows the monthly means for the experimental site during the growing seasons (2020) of the maximum and minimum temperatures (°C), relative humidity (%), wind speed (m/sec), and daily sunshine (hours/day) that were acquired from the Meteorological Station in Assiut, Egypt.

A good stand of plants grew, and then each pot was thinned until five seedlings remained. Seedlings were selected on the basis of their uniform growth rate, and undesired seedlings were removed. Water regime treatments were conducted during the first irrigation at sowing. The drought treatments included three water regimes [well water (100% field water capacity (FC) at 11% water content) as a control and two drought stresses (80 and 60% FC)] that were chosen on the basis of a previous preliminary experiment. Other seeds were soaked in priming agents (ZnO NPs 60 mg/L or ZnO bulk 60 mg/L) and cultivated under the previously mentioned levels of field capacity. Each pot served as a symbol for the experimental unit. The experiment was carried out in six replicates, which were classified as follows:

1. Well water at 100% FC was used as a control.
2. Mildes at 80% FC.
3. Highest level at 60% FC.
4. Unstressed seedlings of those seeds were primed with the previously mentioned concentrations of ZnO NPs or ZnO bulk.
5. Drought-stressed seeds were primed with the previously mentioned concentrations of ZnO NPs or ZnO bulk.

**Table 2:** Average monthly meteorological data of Assiut agrometeological station during the growing seasons.

Year	Month	T max (°C)	T min (°C)	RH %	w.s / km/h	Sunshine (hours)	Solar radiation (Mj/m^2^.d)	ETo (mm/day)
2020	January	19.3	5.8	52.8	13.9	8.9	344	3.5

T Max = Maximum temperature (°C), T min = Minimum temperature (°C), RH = Relative humidity (%), W.S = Wind speed (Km/h) ETo = reference crop evapotranspiration.

### Harvesting and plant growth yield

The plants were separated into roots and shoots at the end of the 20-day experiment. Filter paper was used to gently wipe the roots after they were quickly rinsed with deionized water. The fresh weight (FW) of the shoots and roots was determined, and the plants were immediately stored at -80 °C for further analysis. Another portion of the recently obtained roots and shoots was oven-dried at 70 °C to determine the dry weight (DW).

#### Growth measurement

Plant growth parameters such as shoot height and root length were recorded. After the plant materials were dried in an oven for 72 h at 70 °C, the root and shoot dry weights and water contents were calculated via the following formula: (fresh weight‒dry weight)/fresh weight × 100.

#### Photosynthetic pigments

The determination of photosynthetic pigments (chlorophyll a, b and carotenoids) was conducted using fresh leaves suspended until they became colorless in 10 ml of 95% ethanol at 60 °C. The absorbance readings were determined spectrophotometrically at 663, 644, and 452 nm. The chlorophyll and carotenoid concentrations were calculated as described by Lichthenthale^39^ as mg g^− 1^ FW.

#### Preparation of plant extract (for determination of enzymatic and nonenzymatic antioxidants, in addition to determination of antioxidant potential)

Fresh plant samples (shoots and roots) were ground in a chilled mortar and pestle with 5 ml of buffer solution containing 50 mM Tris HCl (pH 7), 1 mM sodium EDTA and 3 mM MgCl_2_. The extract was subsequently centrifuged at 4 °C for 10 min at 5000 rpm. The resulting supernatant was used to determine enzymatic and nonenzymatic antioxidants, in addition to free-radical scavenging abilities^40^.

##### Enzymatic antioxidants

*Super oxide dismutase (EC 1.15.1.1)*

SOD activity was assayed following the autoxidation of epinephrine (adenochrome) as described by Misra and Fridovich^41^, but with few adjustments, employing 4020 M^− 1^ cm^− 1^ as the extinction coefficient. The activity was evaluated in a final volume of 2 ml of the reaction medium that contained 100 µl of 5.5 mg/ml epinephrine (dissolved in 10 mM HCl, pH 2), 50 µl of protein extraction, 0.1 mM EDTA, and 50 mM sodium carbonate buffer (pH 10.2). At 480 nm, autoxidation of epinephrine was measured for two minutes. The enzyme activity was measured as mM adenochrome min^-1^ mg^-1^.

*Catalase (EC 1.11.1.6)*

Catalase (CAT) activity was assayed according to previous methods Aebi^42^. The reaction medium contained 50 mM potassium phosphate buffer (pH 7), 10 mM H_2_O_2_ and 100 µl of protein extract in a 4 ml volume (for shoots and roots). The decrease in absorbance at 240 nm was used to calculate the activity.

*Peroxidase (EC 1.11.1.7)*

Peroxidase (POD) activity was determined according to Zaharieva et al.^43^, following the formation of tetraguaiacol at A_470_ nm.

*Ascorbate peroxidase (EC 1.11.1.11)*

The method for measuring ascorbate peroxidase (APX) was described previously Jiang and Zhang^44^ but with some modifications. The rate of hydrogen peroxide-dependent oxidation of ascorbic acid was determined in a reaction mixture containing 4 ml of reaction medium containing 5 mM potassium phosphate buffer (pH 7), 1 mM H_2_O_2_ and 40 µl of enzyme extract from shoots and roots. The activity was determined by recording the decrease in A_290_ following the oxidation rate of ascorbic acid for 3 min.

##### Nonenzymatic antioxidants

*Determination of free phenolic compounds*

Phenolics were determined according to Kofalvi and Nassuth^45^ using Folin-Ciocalteu’s phenol reagent. The extracts (100 µL) were mixed with 2.5 ml of 20% Na_2_CO_3_ and 0.5 ml of 2 N Folin-Ciocalteu’s reagent. The sample absorbance was measured at 725 nm using an EMC-11-UV spectrophotometer. The concentration of phenol in the extract was assessed *via* a standard curve prepared with gallic acid and expressed as µg g^− 1^ FW.

*Total flavonoid content*

The total flavonoid content was measured *via* the method described by Moreno et al.^46^ with slight modifications, and the results are expressed as quercetin equivalents. An aliquot of 100 µL of Tris solution containing 1 mg of extract was added to test tubes containing 0.1 ml of 10% aluminum nitrate, 0.1 ml of a 1 M potassium acetate solution and 3.8 ml of methanol. After 40 min at room temperature, the absorbance was measured at 415 nm. Quercetin was used as a standard. The results are presented as µg g^-1^ FW.

*Total antioxidant activity (DPPH)*

The DPPH stable free radical scavenging activity was determined *via* the method described by Blois^47^. A 0.1 mM solution of DPPH in methanol was prepared, and 1 ml of this solution was added to 3 ml of various concentrations (0.2 to 1.0 mg/ml) of sample dissolved in methanol for testing. After 30 min, the absorbance was measured at 517 nm. Ascorbic acid and BHT were used as reference materials. The percentage of inhibition (I) was calculated as the radical scavenging activity as follows:

$${\text{I}} = \left( {{\text{Abs}}\;{\text{ control }} - {\text{ Abs}}\;{\text{ sample}}} \right)/{\text{Abs }}\;{\text{control}} \times 100.$$

*Reducing power*

The process suggested by Oyaizu^48^ was performed with a few modifications to estimate the reducing power of the samples. The mixture containing 0.5 ml of 0.2 M phosphate buffer (pH 6.6) and plant extract (0.1 ml in addition to 0.5 ml of 0.1% K_3_[Fe(CN)_3_] was incubated in a water bath at 50 °C for 20 min. This mixture was centrifuged at 1000 rpm for 10 min after the addition of trichloroacetic acid (0.5 mL). One hundred microliters of 0.01% FeCl_3_ and 1 ml of distilled water were added to 1 ml of the supernatant. After the mixture was incubated for ten minutes at 37 °C, the absorbance was recorded at 700 nm. The data are expressed as mg g^− 1^ FW, with ascorbic acid used as the standard.

*Free radical scavenging abilities (%)*

*Hydroxyl radical* (*OH·*^*-*^) *scavenging percentage*

The assay for scavenging hydroxyl radicals (OH∙^-^) was conducted by Kunchandy and Rao^49^. The absorbance of the plant extracts was measured at A_532_ nm and compared to that of a blank consisting of buffer and deoxyribose. One milliliter of reaction mixture containing 500 µl of previously prepared extracts of different concentrations (3–1000 µg/ml) and 100 µl of each of 2-deoxy-D-ribose (28 mM), EDTA (1.04 mM), FeCl_3_ (0.2 mM) or ascorbic acid (1.0 mM) was incubated at 37 °C for 1 h. The inhibition of deoxyribose was calculated via the following formula: ${\text{I}} = \left( {{\text{Abs}}\;{\text{ control }} - {\text{ Abs}}\;{\text{ sample}}} \right)/{\text{Abs }}\;{\text{control}} \times 100.$

*Hydrogen peroxide (H*_2_ *O*_2_ *) scavenging %*

The percentage of H_2_O_2_ radical scavenging was assayed following the method described by Long et al.^50^ with minor changes. Aliquots of 50 mM H_2_O_2_ and various concentrations (0–2 mg/ml) of the samples were mixed (1:1 v/v) and incubated for 30 min at room temperature. After incubation, 90 µl of the H_2_O_2_ sample mixture was mixed with 10 µl of HPLC-grade methanol, and 0.9 ml of FOX reagent was added (prepared in advance by mixing 9 volumes of 4.4 mM BHT in HPLC-grade methanol with 1 volume of 1 mM xylenol orange and 2.56 mM ammonium ferrous sulfate in 0.25 M H_2_SO_4_). The reaction mixture was then vortexed and incubated at room temperature for 30 min. At 560 nm, the absorbance of the ferric-xylenol orange complex was measured using sodium pyruvate as the standard compound^51^.

*Nitric oxide scavenging %*

Nitric oxide from aqueous sodium nitroprusside (SNP) solution reacts with oxygen at physiological pH to form nitrite ions, which can be detected *via* the Griess‒Illosvoy reaction according to Garratt^52^. Sodium nitroprusside (1 ml of 10 mM) was mixed with 1 ml of previously prepared extract at different concentrations (3–110 µg/ml) in phosphate buffer (pH 7.4). The mixture was incubated at 25 °C for 150 min. To 1 ml of the incubated mixture, 1 ml of Griess’ reagent (1% sulphanilamide, 2% o-phosphoric acid and 0.1% naphthyl ethylene diamine dihydrochloride) was added. Using spectrophotometry, the pink chromophore produced by diazotizing nitrite ions with sulfanilamide and then coupling with NED was identified at 540 nm compared with that of a blank sample. Curcumin was used as the standard.

*Metal chelation*

The metal chelating ability was determined following the method of Decker and Welch^53^ with minor modifications. A total of 1.6 ml of each extract stock solution (0.2, 0.4, 0.6, 0.8 or 1 mg/ml) was mixed with 2.16 ml of distilled water and 80 µl of 2 mM FeCl_2_ in a test tube. The reaction was initiated by the addition of 160 µl of 5 mM ferrozine. The solutions were mixed well and allowed to stand for 10 min at room temperature. After incubation, the absorbance was measured at 562 nm spectrophotometrically. Distilled water (1.6 ml) instead of sample solution was used as a control. Distilled water (160 µl) instead of ferrozine solution was used as a blank, which was used for error correction because of the unequal color of the sample solutions. L-ascorbic acid was used as a reference standard. All measurements were performed in triplicate. The ferrous ion-chelating ability was calculated as follows:

$$\% {\text{ scavenging }}\;{\text{activity}}\;\left( {{\text{ferrous}}\;{\text{ ion }}\;{\text{chelating }}\;{\text{ability}}} \right) = \frac{{\left( {{\text{Ac}} - {\text{Ae}}/{\text{As}}} \right)}}{{{\text{Ac}}}} \times 100$$

where Ac is the absorbance of the control reaction; Ae is the absorbance of the plant extract; and As is the absorbance of the standard.

*Lipid peroxide inhibition %*

The percentage of lipid peroxidation inhibition was assayed according to the Janero^54^ method. TBA reacts with MDA to form a diadduct, a red chromogen. Shoot and root homogenates were prepared at − 5 °C with 5 mM phosphate-buffered saline (PBS) (1:10) for 30 min. The homogenate was centrifuged for 15 min, and a clear cell-free supernatant was used for the study of in vitro lipid peroxidation. A total of 0.1 mL of each extract at different concentrations (10–2000 µg/mL) in dimethyl sulfoxide was added to the test tube. A total of 1.4 mL of 50 mM PBS buffer and 0.5 mL of plant tissue homogenate were added to the test tubes. After incubation at 37 °C for 15 min, the reaction was stopped by the addition of 1 mL of 10% TCA and 1 mL of 0.8% TBA. The mixture was then heated at 100 °C for 15 min. The samples were cooled and centrifuged, and the absorbance of the supernatants was measured at 532 nm. The percentage inhibition of lipid peroxidation was calculated as the percentage of inhibition (I) and recorded *via* the following formula:

$${\text{I}} = \left( {{\text{Abs}}\;{\text{ control }} - {\text{ Abs}}\;{\text{ sample}}} \right)/{\text{Abs }}\;{\text{control}} \times 100.$$

The absorbance of the upper organic layer was measured at A532.

##### Primary metabolites

*Soluble carbohydrates*

Carbohydrate contents were determined via the anthrone sulfuric acid technique^55,56^. The anthrone-sulfuric acid reagents prepared from 0.2 g of anthrone, 30 ml of distilled water, 8 ml of absolute ethyl alcohol, and 100 ml of concentrated H_2_SO_4_ were mixed in a conical flask under continuous cooling in an ice bath. This reagent was freshly prepared. The sample extract (0.2 ml) and 4.5 ml of anthrone reagent were thoroughly mixed and boiled in a water bath for 7 min; thereafter, the mixture was directly cooled under tap water. A calibration curve was constructed using pure glucose. The developed blue–green color was read at 620 nm in 3 ml and compared with a blank containing only anthrone reagent. The soluble carbohydrate content was expressed as mg g ^[-1^ FW.

*Free amino acids*

The method of Moore and Stein^57^ was used to determine the free amino acid content of the crude extract. The shoot and root extracts (0.2 ml) and 1 ml of SnCl_2_ reagent (10 mg of SnCl_2_ was dissolved in 10 ml of citrate buffer and 10 ml of ninhydrin reagent) were thoroughly mixed, boiled in a water bath for 20 min and cooled to room temperature. Five milliliters of diluent solvent was added to the mixture and mixed well, after which the absorbance was measured at 570 nm. However, with this method, traces of proline and hydroxylproline can be encountered. Glycine was used to generate a calibration curve, and the free amino acid concentration was calculated as mg g ^− 1^ FW.

*Soluble proteins*

Folin reagent was used to determine the soluble protein content following the methods of Lowry et al.^58^. Five millilitres of the alkaline reagent solution was added to 0.05 ml of the shoot and root extracts and mixed. The reaction mixture was mixed rapidly with 0.5 ml of diluted Folin–Ciocalteu’s reagent (1: 2 v/v) and left for 30 min, after which the absorbance was measured against an appropriate blank at 750 nm. The soluble protein content was expressed as mg g^− 1^ FW and bovine serum albumin (BSA) was used to prepare a calibration curve.

#### Statistical analysis

Data were collected in triplicate from six measurements out of two independent factorial experiments. Analysis of variance (ANOVA) was conducted via the SPSS statistical package (version 11.0). The comparison of the means for significant differences was performed via Duncan’s multiple range test at *p* *≤* 0.05 as a post hoc test. All the assessed attributes were analyzed via principal component analysis (PCA) variance regression ordination. The heatmap and scatter plot were generated *via* ggplot packages and visualization of the corrplot, which was integrated into R software (RStudio). The data (mean values) were normalized to a standard range of ± 1 to perform the analysis.

## Results

### Combining experimental (FTIR) and simulated TD-DFT IR for the nanoblend and nanocomposite

Figure 1A shows the FTIR spectrum of the ZnO NPs in the 400–4000 cm^− 1^ range. Three absorption bands appeared at 3438, 1643 and 447 cm^− 1^. The peak at 3438 cm^− 1^ can be assigned to the stretching and bending vibrations of OH groups of absorbed water molecules during the measurements. The other absorption band at 1643 cm^− 1^ in the FTIR spectrum of our samples is also correlated with the ZnO nanoparticles. The band at 447 cm^− 1^ is assigned to the stretching vibration of Zn–O in the octahedral coordination. Gaussian 09 W software at the WBX97XD is utilized to simulate the infrared (IR) computations for the isolated zinc oxide nanoparticle ZnO HNB/Iso gaseous phase matrices at 6–311 G/TD-DFT. The TD-DFT-Gaussian 09 W vibration values exhibit a high degree of similarity to the values observed in the experimental data. To determine the spectroscopic characteristics of the gaseous-phase ZnO HNB/Iso of the isolated molecule, the theoretical IR spectrum was evaluated. In Figs. A and C the minute discrepancies between the predicted and measured frequencies are illustrated. The fundamental difference lies in the fact that the count was performed under vacuum conditions, whereas the measurements were optimized for solid-state conditions.

### Combining the experimental (XRD) and simulated polymorph tools XRD for zinc oxide nanocomposite

The structural properties of the ball-milling technique-prepared ZnO NPs were investigated *via* X-ray diffraction. The XRD pattern of the ZnO NPs is shown in Fig. 1B It shows well-defined peaks with orientations in the (111), (200), (202), (311), (222), (400), (313), (402), (422), and (511) planes. The absence of impurity peaks suggests the formation of pure and single-phase zinc oxide. The lattice parameters (a, b, and c) for the cubic phase of the ZnO NPs were calculated *via* the formula $\:(\frac{a+b}{2}/\frac{b+c}{2}/\frac{a+c}{2}\::a:a:a):2/3$, and their values were found to be a = b = c = 4.214 Å. The calculation yields $\:{D}_{Av}$ values in the range of 1.81–33.71 nm with an average size, $\:{D}_{av}\cong\:22.69\:nm$, are given in Table 2. The specific surface area (A) of the ZnO NPs is related to $\:{D}_{Av}$ *via* the following formula: $\:(A=6/\rho\:{D}_{Av})$, where $\:\rho\:$ is the density of the ZnO NPs (5.61 g/cm^3^). With this equation, $\:{A}_{av}=13.160$ m^2^/g. The XRD patterns of the pure ZnO NPs are depicted in Fig. 1. From the XRD pattern, two main peaks occur at hkl 111 and 202, and another weak peak occurs at hkl 402. These findings indicate that the crystalline structure of the ZnO NPs is spherical with severe aggregation (Table 3).

**Table 3:** The experimental and calculated XRD data using the refine Version 3.0 Software Program (Kurt Barthelme’s & Bob Downs) for the simulated ZnO NPs/Iso and experimental patterns for ZnO NPs.

Sample	2 Theta	*P*.H.	hkl	d (Å)	Debye–Scherrer		A×10^2^
					(β)	D_av_	
ZnO NPs	11.91	618.1	111	2.433	0.2962	28.19	3.7945
DC = 1,000,424	14.59	507.0	200	2.1070	0.2524	33.17	3.2246
Crystal form is Cubic	16.43	1000	202	1.4899	0.3981	21.07	5.0749
Space group = F23 (196)	27.73	327.6	311	1.2706	0.2537	33.71	3.1724
a = b = c = 4.214 (Å)	36.82	407.3	222	1.2165	0.3369	2.60	41.172
α = γ = β = 90^o^	43.12	275.7	400	1.0535	0.4929	1.81	59.044
V = 20,000(5814)	46.64	73.2	313	0.9668	0.3639	24.85	4.3041
Rmse = 0.02406456852	48.23	367.6	402	0.9423	0.3320	27.40	3.9030
ME= -27.340	49.30	188.6	422	0.8602	0.3332	27.42	3.9005
	57.18	630	511	0.8110	0.3545	26.68	4.0092
Average						22.69	13.160

The XRD calculations for zinc oxide nanoparticles were performed via the Debye–Scherrer method within the range of 5 ≤ 2θ ≤ 60, with $\:1/dhkl\:=\:0.0566\:{A˚}^{-1}-\:0.7446\:{A˚}^{-1}$, λ = 1.540562 Å, $\:{I}_{2}/{I}_{1}=0.5$, polarization = 0.5, and the Pesedo-Voigt function. Polymorph, via Content Studio software (version 7.0), was used to compute the theoretical X-ray diffraction models (refer to Fig. 1). The integrals in the Brillouin zone were 2 × 2 × 1 (polymorph ZnO NPs) and 4 × 4 × 2 (polymorph nanoblend and nanocomposite), as shown in the inset of Fig. 1.

### The growth parameters

The results in Table 4 indicate that decreasing the field capacity significantly affected the fresh and dry weights as well as the lengths of the shoots and roots of the wheat seedlings. The seedlings grown at 60% field capacity presented the lowest values of fresh weight, dry weight and length for shoots (63, 58, and 54%, respectively). Additionally, the same previously mentioned conditions caused drastic effects on the same parameters of the roots (39, 50.5, and 60%, respectively) compared with those of the control seedlings. However, priming seeds with either ZnO NPs or bulk ZnO under different drought conditions (80% and 60% FC) mitigated the negative effects of drought stress. There was no significant effect on shoot dry weight in seedlings grown at 60% FC, except for those primed with ZnO NPs or bulk ZnO.

**Table 4:** Effects of the priming agents (ZnO NPs 60 mg L^− 1^ or ZnO bulk 60 mg L^− 1^) under different levels of drought (100%, 80%, and 60% of field capacity) on length, fresh weight (Fwt.), dry weight (dwt.) In shoots and roots and photosynthetic pigments (Chlorophyll a = Chl. A, Chlorophyll b = chl. B and carotenoids = carot.) In leaves of wheat seedlings.

	Drought treatments	Length (Cm)		Fwt. (g/ plant)		Dwt. (g/ plant)		Photosynthetic pigments (mg g^− 1^ Fwt)		
	(FC)	Shoot	Root	Shoot	Root	Shoot	Root	Chl. a	Chl. b	Carot.
Cont	100%	16.2 ± 0.6^c^	18.3 ± 0.3^d^	0.22 ± 0.0^c^	0.51 ± 0.0^d^	0.03 ± 0.00^b^	0.04 ± 0.00^e^	0.88 ± 0.00^cd^	0.64 ± 0.00^e^	0.67 ± 0.00^e^
	80%	11.9 ± 0.2^b^	14.5 ± 0.3^c^	0.15 ± 0.0^a^	0.22 ± 0.01^a^	0.02 ± 0.00^a^	0.03 ± 0.00^b^	0.86 ± 0.00^c^	0.49 ± 0.00^c^	0.59 ± 0.01^cd^
	60%	8.8 ± 0.8^a^	11.0 ± 0.6^a^	0.14 ± 0.00^a^	0.20 ± 0.01^a^	0.02 ± 0.00^a^	0.02 ± 0.00^a^	0.67 ± 0.00^b^	0.46 ± 0.00^c^	0.49 ± 0.00^b^
ZnONPs 60 mg L^− 1^	100%	18.5 ± 0.2^c^	17.0 ± 0.0^d^	0.33 ± 0.17^d^	0.41 ± 0.01^c^	0.04 ± 0.00^c^	0.03 ± 0.00^c^	0.66 ± 0.00^b^	0.32 ± 0.00^a^	0.44 ± 0.00^b^
	80%	17 ± 0.2^c^	14.0 ± 0.0^bc^	0.30 ± 0.02^d^	0.22 ± 0.00^a^	0.03 ± 0.00^b^	0.04 ± 0.00^d^	0.92 ± 0.01^cd^	0.47 ± 0.00^c^	0.63 ± 0.00^de^
	60%	13.7 ± 0.4^b^	14.0 ± 0.0^bc^	0.17 ± 0.2^b^	0.27 ± 0.00^b^	0.02 ± 0.00^a^	0.03 ± 0.00^b^	0.76 ± 0.19^b^	0.47 ± 0.01^c^	0.55 ± 0.17^c^
ZnO bulk 60 mg L^− 1^	100%	18.6 ± 0.5^c^	21.2 ± 0.4^e^	0.21 ± 0.0^bc^	0.42 ± 0.01^c^	0.04 ± 0.00^bc^	0.04 ± 0.00^de^	0.41 ± 0.01^a^	0.397 ± 0.00^b^	0.32 ± 0.14^a^
	80%	17.5 ± 0.6^c^	18.0 ± 0.6^d^	0.21 ± 0.01^bc^	0.30 ± 0.00^b^	0.04 ± 0.00^bc^	0.04 ± 0.00^e^	1.12 ± 0.07^e^	0.55 ± 0.01^d^	0.6 ± 0.03^cde^
	60%	12.0 ± 0.6^c^	12.7 ± 0.3^b^	0.16 ± 0.01^ab^	0.19 ± 0.14^a^	0.02 ± 0.00^a^	0.02 ± 0.00^a^	0.98 ± 0.00^d^	0.48 ± 0.00^c^	0.63 ± 0.00^de^

The data are means of six replicates ± SD. Different letters indicate statistically significant differences according to Tukey’stest (*p* ≤ 0.05).

Similar responses to different drought levels (80% and 60% FC) were observed for the Chl *a*, Chl *b* and carotenoid concentrations in stressed seedlings, with significant reductions of 77%, 72% and 73%, respectively, in the 60% FC treatment compared with the control. Seed priming with ZnO NPs or ZnO bulk had a positive effect on the photosynthetic pigment concentrations in the leaves of stressed plants at both 80% and 60% FC. However, there was no significant effect on the Chl *a* concentration in the leaves of plants grown at 60% FC when primed with ZnO NPs or bulk ZnO.

### Antioxidant system

#### Enzymatic antioxidants

##### Ascorbate peroxidase (APX)

**Fig. 2:**
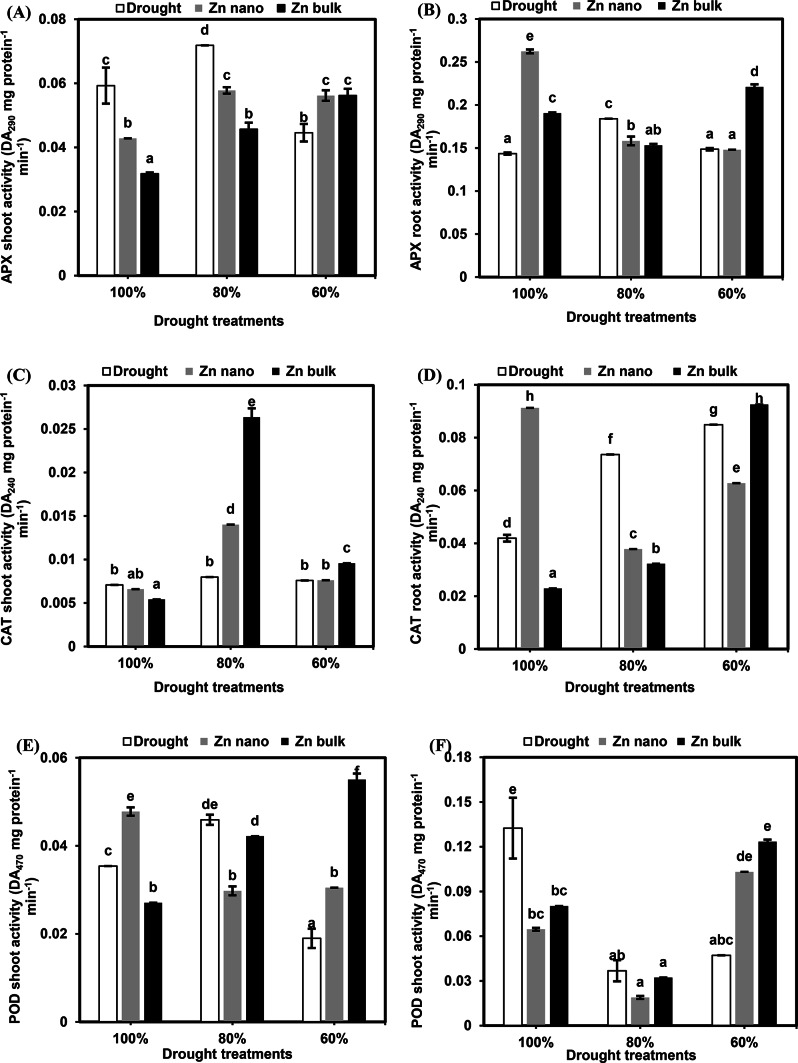

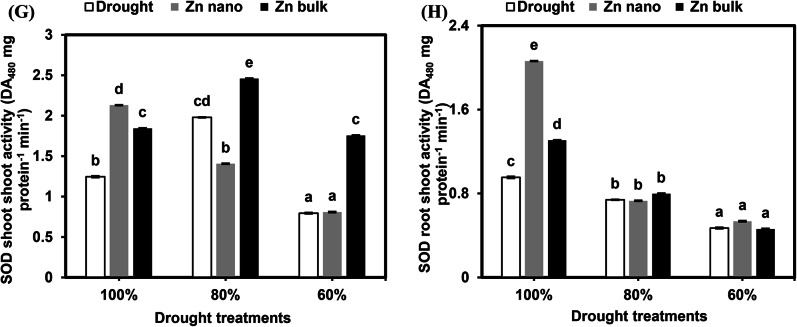
Effects of the priming agents (ZnO NPs 60 mg L^− 1^ or ZnO bulk 60 mg L^− 1^) under different levels of drought (100%, 80%, and 60% of field capacity) on ascorbate peroxidase (APX; **A** and **B**), catalase (CAT; **C** and **D**), peroxidase (POD; **E** and **F**), and superoxide dismutase (SOD; **G** and **H**) activities in shoot and root of wheat seedlings. The data are means of six replicates ± SD. Different letters indicate statistically significant differences according to Tukey’stest (*p* ≤ 0.05).

A recent study examined the impact of drought stress on ascorbate peroxidase (APX) activity in the shoots and roots of wheat seedlings (Fig. 2A and B). Under 80% FC drought stress, APX activity increased by 121% for shoots and 128% for roots, whereas severe stress at 60% FC led to a 75% decrease in APX activity in shoots compared with that of the controls. Under 80% FC, the priming of seeds with ZnO NPs or ZnO bulk reduced APX activity to 80% and 63.5% in shoots and 47% and 62.6% in roots, respectively. However, under severe stress at 60% FC, priming with ZnO NPs or ZnO bulk significantly increased APX activity to 126% in shoots and 112% in roots, respectively, compared with that in stressed plants without priming.

##### Catalase (CAT)

The catalase (CAT) activity in wheat seedlings under drought stress or priming with ZnO NPs or bulk ZnO is shown in Fig. 2C and D. Drought stress at 80% and 60% FC increased the CAT activity in roots more than it did in shoots by 175% and 202%, respectively. Compared with stressed seedlings, these seedlings grown at 80% FC with ZnO NPs or with ZnO bulk priming presented greater CAT activity in shoots by 175% and 300%, respectively. However, compared with stressed seedlings, ZnO NP priming at 60% FC inhibited CAT activity in roots and reduced it to 73.9%. Conversely, compared with stressed seedlings, ZnO bulk priming at 60% FC increased the CAT activity in shoots and roots by 126% and 108%, respectively.

##### Peroxidase (POD)

Severe drought stress (60% FC) was found to reduce peroxidase enzyme activity in shoots by 53.6%. Similarly, compared with those in drought-stressed plants, enzyme activity in roots decreased significantly at 80% and 60% FC, with values of 27.7% and 35.6%, respectively. Compared with nonprimed stressed seedlings, priming seeds with ZnO NPs or ZnO bulk increased enzyme activity in drought-stressed plants (60% FC), with values of 91.8% and 289.9% for shoots and 218.6% and 261.6% for roots, respectively (Fig. 2E and F).

##### Super oxide dismutase (SOD)

The results of the present study revealed that drought stress at 80% FC increased SOD activity in shoots by 58%, whereas severe stress at 60% FC decreased SOD activity by 63.6%. In the roots, 80% and 60% FC led to significant reductions in SOD activity of 77.6% and 49.4%, respectively. Priming seeds with bulk ZnO increased SOD activity in shoots under drought stress at 80% and 60% FC by 124% and 220.7%, respectively. However, priming with ZnO NPs or ZnO bulk did not affect SOD activity in roots under different drought stress levels (Fig. 2G and H).

#### Nonenzymatic antioxidants

##### Free phenolic compounds

**Fig. 3:**
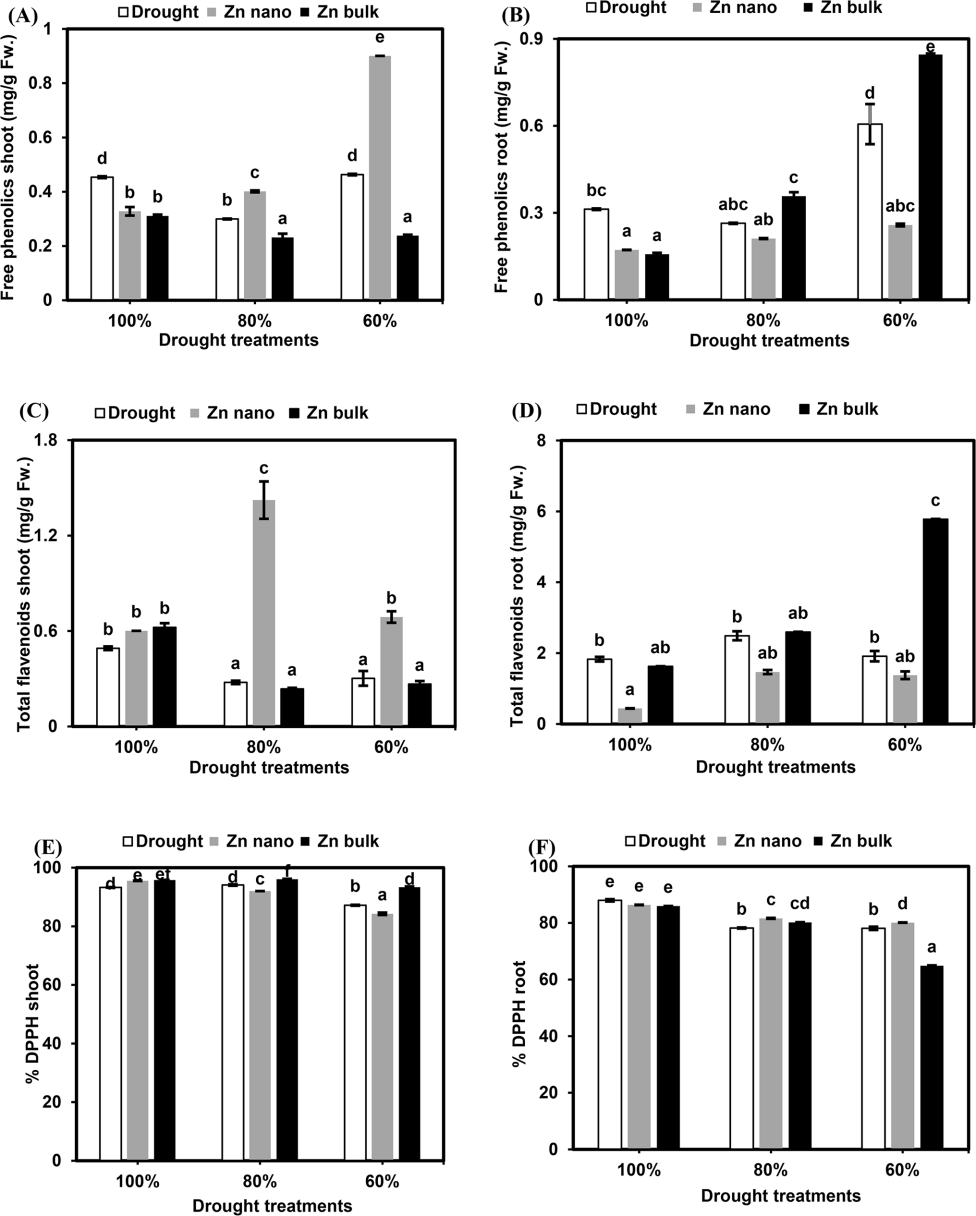

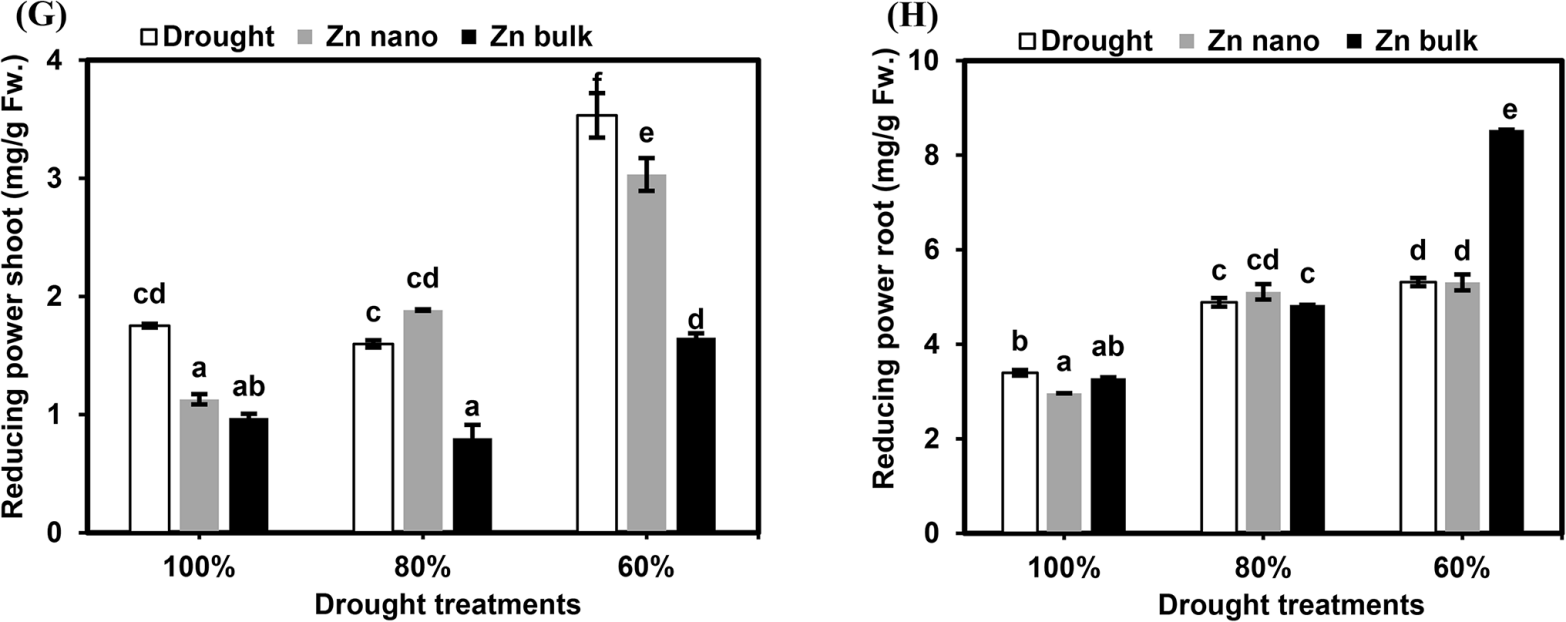
Effects of the priming agents (ZnO NPs 60 mg L^− 1^ or ZnO bulk 60 mg L^− 1^) under different levels of drought (100%, 80%, and 60% of field capacity) on phenolics (**A** and **B**), flavenoids (**C** and **D**), Total antioxidant activity (DPPH; **E** and **F**), and reducing power (**G** and **H**) in shoot and root of wheat seedlings. The data are means of six replicates ± SD. Different letters indicate statistically significant differences according to Tukey’stest (*p* ≤ 0.05).

The concentrations of free phenolics in the shoots and roots of wheat seedlings grown under drought stress alone or in combination with seed priming with ZnO NPs or ZnO bulk are shown in Fig. 3A and B. Under drought conditions, the accumulation of free phenolics in shoots was significantly inhibited at 80% FC to 66%, whereas no significant effects were observed at severe drought levels (60% FC). Conversely, under the same conditions, the concentration of free phenolics in the roots increased significantly (193.8%) at the severe drought level (60% FC) compared with that in the control plants. Seed priming with ZnO Nps only increased the concentration of free phenolics in the shoots at 80% and 60% FC (133.9% and 194.4%, respectively). Similarly, seed priming with bulk ZnO at 80% and 60% FC increased phenolic concentrations in roots to 135.4% and 1139.6%, respectively, compared with those in stressed seedlings without priming.

##### Total flavonoids

The total flavonoid concentrations of wheat seedling shoots and roots under drought stress alone or in combination with seed priming with ZnO NPs or ZnO bulk are shown in Fig. 3C and D. Drought stress at 80% and 60% FC significantly reduced shoot concentrations by 56.3% and 61.5%, respectively, whereas no significant effects were detected in the roots. Compared with stress, seed priming with ZnO NPs under 80% and 60% FC stress increased shoot concentrations by 413.3% and 127.4%, respectively. Similarly, seed priming with bulk ZnO increased their concentrations in the roots by 201.8% at the 60% FC stress level compared with stressed seedlings.

##### Total antioxidant activity (DPPH)

The impacts of drought stress, either alone or in combination with seed priming with ZnO NPs or ZnO bulk, on the total antioxidant activity (DPPH) concentrations in the shoots and roots of the tested plants are illustrated in Fig. 3E and F. Severe drought stress at 60% FC significantly reduced the accumulation of antioxidants in the shoots by 93.4%. Similarly, their concentrations in roots were more affected at 80% and 60% FC, decreasing by 88.9% and 88.7%, respectively, compared with control seedlings.

Under stress conditions at 80% and 60% FC, seed priming with ZnO NPs led to significant decreases in total antioxidant activity (DPPH) accumulation in shoots of 97.8% and 96.7%, respectively. However, their concentrations in roots were slightly greater than those stressed seedlings. Conversely, seed priming with bulk ZnO under stressed conditions at 80% and 60% FC increased shoot concentrations to 102.1% and 107%, respectively. However, under severe stress conditions (60% FC), ZnO bulk priming inhibited root concentrations to 83% compared with stressed seedlings without priming.

##### Reducing power

The results of the reducing power in the shoots and roots of wheat seedlings grown under different levels of drought combined with priming with ZnO NPs or bulk ZnO are shown in Fig. 3G and H. Compared with those of control seedlings, the shoot concentrations of drought-susceptible seedlings (60% FC) significantly increased. Similarly, their concentrations in roots increased at 80% and 60% FC by 143.9% and 156.5%, respectively. Seed priming with ZnO NPs under severe stress (60% FC) inhibited 85.8% of shoot priming but did not affect the root concentration. Seed priming with bulk ZnO at 80% and 60% FC reduced the shoot concentration to 50% and 46.7%, respectively. Compared with stress, seed priming with bulk ZnO at 60% FC resulted in greater reducing power accumulation (160%) in the roots.

#### Free radical scavenging abilities

The results of the scavenging abilities of some free radicals, such as hydroxyl radicals (OH^∙−^), hydrogen peroxide (H_2_O_2_), nitric oxide (NO), and metal chelation, and the inhibition of lipid peroxidation are presented in Table 5.

**Table 5:** Effects of the priming agents (ZnO NPs 60 mg L^− 1^ or ZnO bulk 60 mg L^− 1^) under different levels of drought (100%, 80%, and 60% of field capacity) on hydroxyl radical scavenging (% OH.^−^), hydrogen peroxide scavenging (% H_2_O_2_), nitric oxide scavenging (% NO), percentage of metal chelating (% MEC) and inhibition of lipid peroxide (% MDA) in shoots and roots of wheat seedlings.

	Drought treatments	Scavenging % OH.^−^		Scavenging H_2_O_2_ (mg/g Fwt.)		Scavenging NO%		% MEC		% inhibition of lipid peroxidation	
	(FC)	Shoot	Root	Shoot	Root	Shoot	Root	Shoot	Root	Shoot	Root
Cont	100%	93.3 ± 0.1^d^	88.0 ± 0.4^f^	77.5 ± 0.4^d^	59.3 ± 0.7^c^	95.4 ± 0.1^ef^	88.7 ± 0.4^f^	93.8 ± 0.1^b^	94.0 ± 0.1^e^	76.6 ± 1.3^c^	18.5 ± 0.2^ab^
	80%	94.1 ± 0.2^e^	78.2 ± 0.2^b^	76.5 ± 0.0^cd^	44.3 ± 0.7^b^	94.5 ± 0.2^e^	82.6 ± 0.2^c^	94.5 ± 0.2^b^	90.2 ± 0.4^b^	74.7 ± 1.4^c^	54.9 ± 0.9^e^
	60%	87.2 ± 0.2^b^	78.1 ± 0.5^b^	55.3 *+ 0.7*^b^	70.8 ± 6.0^e^	88.1 ± 0.2^b^	81.7 ± 0.6^b^	91.0 ± 0.4^a^	92.0 ± 0.2^c^	18.2 ± 4.2^a^	21.8 ± 4.4^b^
ZnO NPs 60 mg L^− 1^	100%	95.5 ± 0.1^f^	86.4 ± 0.9^e^	84.6 ± 0.7^e^	66.7 ± 1.3^d^	96.0 ± 0.1^f^	88.3 ± 0.1^f^	97.4 ± 0.1^d^	94.7 ± 0.2^ef^	86.1 ± 2.3^c^	54.6 ± 1.9^e^
	80%	92.0 ± 0.1^c^	81.6 ± 0.2^d^	74.5 ± 0.6^c^	91.2 ± 4.4^f^	91.2 ± 0.0^c^	83.7 ± 0.2^de^	95.6 ± 0.1^c^	93.2 ± 0.0^d^	76.0 ± 1.3^c^	43.2 ± 0.4^d^
	60%	84.3 ± 0.4^a^	80.1 ± 0.08^c^	50.5 ± 0.6^a^	88.5 ± 0.8^f^	86.6 ± 0.4^a^	83.4 ± 0.1^cd^	91.5 ± 0.0^a^	92.4 ± 0.1^c^	58.4 ± 6.2^b^	40.3 ± 4.9^cd^
ZnO bulk 60mg L^− 1^	100%	95.8 ± 0.3^f^	86.0 ± 0.7^e^	85.8 ± 0.4^ef^	61.1 ± 0.2^cd^	96.8 ± 0.0^f^	89.1 ± 0.1^f^	97.3 ± 0.1^d^	95.3 ± 0.0^f^	81.0 ± 5.5^c^	29.6 ± 4.2^bc^
	80%	96.1 ± 0.1^f^	80.2 ± 0.4^c^	87.0 ± 0.4^f^	65.4 ± 0.3^d^	96.1 ± 0.0^f^	84.4 ± 0.4^e^	97.2 ± 0.4^d^	92.3 ± 0.1^c^	87.3 ± 1.8^c^	9.2 ± 0.2^a^
	60%	93.3 ± 0.1^d^	64.9 ± 0.4^a^	74.9 ± 0.4^c^	29.2 ± 2.0^a^	92.7 ± 0.1^d^	67.9 ± 0.4^a^	96.1 ± 0.0^c^	84.0 ± 0.2^a^	78.9 ± 1.7^c^	7.3 ± 0.2^a^

The data are means of six replicates ± SD. Different letters indicate statistically significant differences according to Tukey’stest (*p* ≤ 0.05).

##### Hydroxyl radical scavenging (OH^.−^ %)

The shoots of drought-stressed seedlings presented a notable decrease in hydroxyl radical scavenging (OH^.−^%) at the severe drought level (60% FC), with inhibition reaching 93.5%. Compared with that of the control seedlings, the concentration of (OH^.−^%) in the roots decreased to 88.9% and 88.8% at 80% and 60% FC, respectively. Seed priming with ZnO NPs under stressed conditions at 80% and 60% FC reduced the (OH^.−^%) to 96.3% and 88.2%, respectively, in the shoots. Priming with ZnO bulk at 80% and 60% FC increased (OH.^.−^%) to 102% and 107%, respectively, in shoots and 102% in roots. However, at 60% FC, the roots significantly decreased by 83% compared with that of stressed seedlings without priming.

##### Hydrogen peroxide (H_2_O_2_) scavenging percentage

The shoots of drought-stressed seedlings presented a significant decrease (71.4%) in hydrogen peroxide (H_2_O_2_) scavenging at severe drought conditions (60% FC). Similarly, compared with the control seeldings, mild drought stress (80% FC) significantly reduced root accumulation (74.7%). Seed priming with ZnO NPs under stress conditions at 80% and 60% FC slightly decreased shoot accumulation. However, the roots of these plants under the same conditions presented a significant increase in accumulation (205% and 124.9%, respectively) compared with those of stressed seedlings without priming. Seed priming with bulk ZnO under stress conditions under severe drought conditions (60% FC) promoted their accumulation in shoots and roots to 135.4% and 147.6%, respectively. However, under mild drought stress (80% FC), roots presented a decrease in accumulation (41.1%) compared with stressed seedlings without priming.

##### Nitric oxide (NO) scavenging %

Drought stress at the highest level (60% FC) resulted in the lowest accumulation of NO% in the shoots of stressed seedlings (88%). Compared with the control, the different levels (80% and 60% FC) slightly reduced the percentage in the roots to 93.1% and 92.1%, respectively. Seed priming with ZnO NPs under stressed conditions at 80% and 60% FC significantly decreased the percentage in the shoots to 96.5% and 98.3%, respectively, while increasing the percentage in the roots to 101.3% and 102.2% at 80% and 60% FC, respectively, compared with stressed seedlings without priming. Seed priming with bulk ZnO under stressed conditions at 80% and 60% FC had stimulating effects on their percentages in shoots to 101.7% and 105.2%, but in roots, only 80% FC promoted their percentages to 102.2%. However, at the severe level of 60% FC, the percentage in the roots decreased to 83.1% compared with that in the stressed seedlings without priming.

##### Metal chelation (MEC %)

Severe drought stress (60% FC) significantly inhibited the percentage of metal chelation (% MEC) in the shoots, with a 97% reduction. In the roots, reductions of 96% and 97.3% were observed at 80% and 60% FC, respectively, compared with those of the controls. Seed priming with ZnO NPs under stressed conditions at 80% FC slightly increased their percentages in both shoots and roots (101.2% and 103.2%, respectively). Similarly, shoots stimulated with bulk ZnO under stressed conditions at 80% and 60% FC (102.1% and 105.6%, respectively), but only 80% FC had stimulatory effects on 102.2% of the roots. However, at the severe level of 60% FC, a significant reduction was observed in the roots (91.3%) compared with the stressed seedlings without priming.

##### Inhibition of lipid peroxidation%

Drought stress, especially at 60% FC, significantly suppressed the percentage of lipid peroxidation inhibition in the shoots of stressed seedlings by 23.3%. In contrast, the roots were stimulated at 80% and 60% FC, with increases of 296.2% and 117.9%, respectively, compared with those of the control seedlings. Seed priming with ZnO NPs at 60% FC increased the percentage of lipid peroxidation inhibition in shoots by 320.6%. Compared with nonprimed stressed seedlings, roots presented varied responses, with a decrease of 78.6% at 80% FC and an increase of 184.8% at 60% FC. Priming with bulk ZnO at 60% FC resulted in the highest percentage of lipid peroxidation inhibition in shoots (433%). In contrast, the percentage of roots decreased to 16.6% and 33.3% at 80% and 60% FC, respectively, compared with that of stressed seedlings without priming.

#### Primary metabolites

##### Soluble proteins

**Fig. 4:**
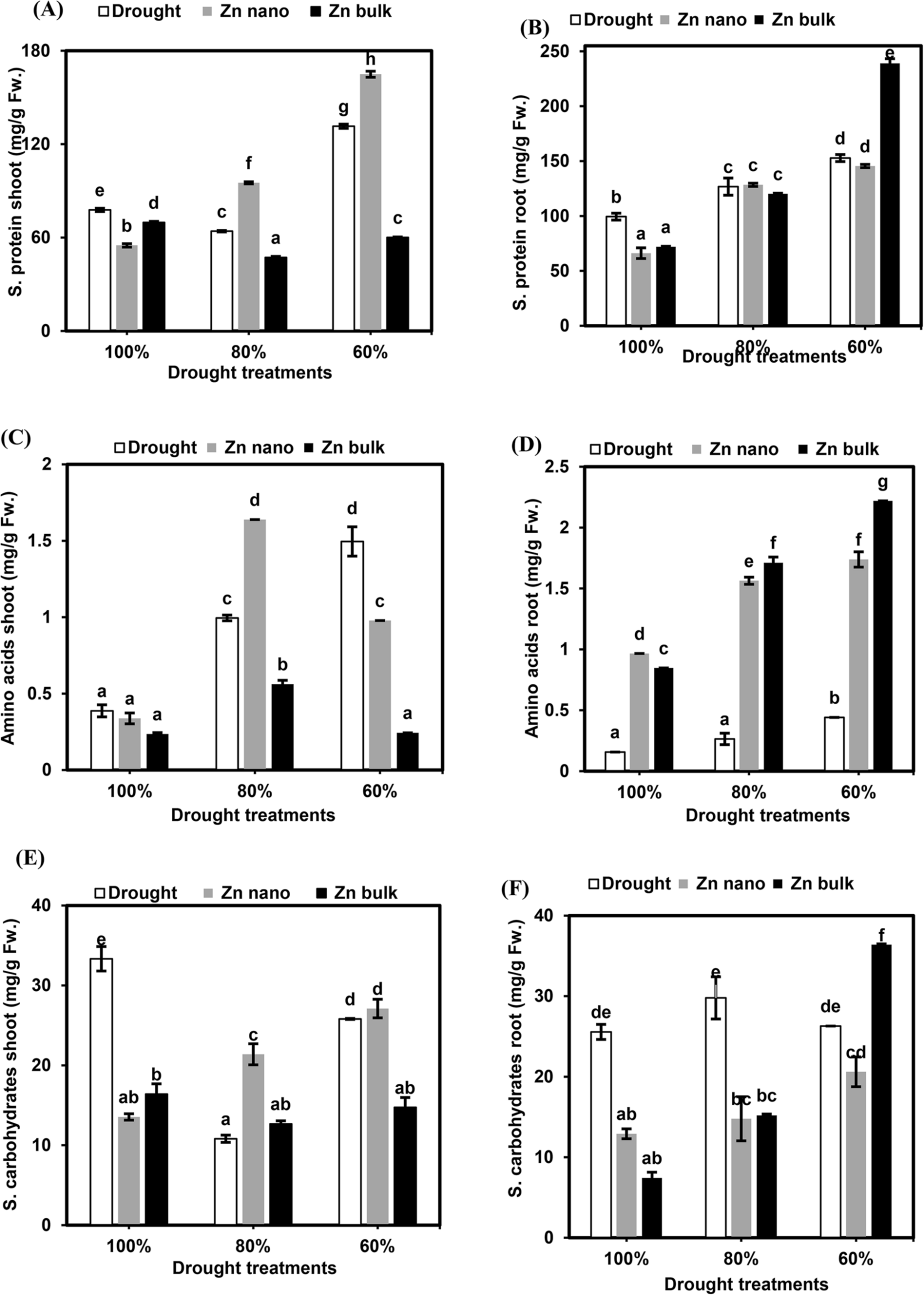
Effects of the priming agents (ZnO NPs 60 mg L^− 1^ or ZnO bulk 60 mg L^− 1^) under different levels of drought (100%, 80%, and 60% of field capacity) on soluble protein (**A** and **B**), amino acids (**C** and **D**) and soluble carbohydrates (**E** and **F**) in shoot and root of wheat seedlings. The data are means of six replicates ± SD. Different letters indicate statistically significant differences according to Tukey’stest (*p* ≤ 0.05).

The results of the soluble protein concentration in the shoots and roots of wheat seedlings under drought stress alone or in combination with priming agents (ZnO NPs or ZnO bulk) are shown in Fig. 4A and B. Drought stress at 80% FC reduced the protein concentration in shoots by 82.4%, whereas at 60% FC, it significantly increased it by 163.1%. Moreover, compared with those of the control seedlings, the roots of the stressed seedlings presented significant increases in soluble protein accumulation at 80% and 60% FC (127.5% and 153.7%, respectively).

The priming of seeds with ZnO NPs under stressed conditions at 80% and 60% FC resulted in stimulatory effects on shoot protein concentrations (148.5% and 125.4%, respectively), whereas root protein concentrations did not significantly change compared with those of stressed seedlings without priming. Compared with non priming stressed seedlings, priming seeds with bulk ZnO under stressed conditions at 80% and 60% FC decreased shoot protein accumulation (73.7% and 45.7%, respectively); conversely, at 60% FC, it promoted root protein accumulation (156.4%).

##### Free amino acids

The results of the free amino acid concentration in the shoots and roots of wheat seedlings under different levels of drought stress, with or without seed priming agents (ZnO NPs or ZnO bulk), are shown in Fig. 4C and D. Drought stress at 80% and 60% FC increased free amino acid accumulation in shoots and roots (256.9% and 386.1%, respectively), with a significant increase in roots at 60% FC (281.2%) compared with control seedlings. Seed priming with ZnO NPs under 80% FC stress increased accumulation in shoots (164.6%), whereas, at 60% FC, it reduced accumulation (65.4%). Different drought levels (80% and 60% FC) combined with ZnO NPs priming significantly increased root accumulation (590.5% and 393.8%, respectively) compared with stressed seedlings without priming. Compared with no priming, ZnO bulk priming under 80% and 60% FC stress decreased amino acid accumulation in shoots (56.4% and 16.3%, respectively) but increased amino acid concentrations in roots to 646.1% and 502.8%, respectively.

##### Soluble carbohydrates

The concentrations of soluble carbohydrates in the shoots and roots of wheat seedlings under various drought stress conditions, with or without seed priming with ZnO NPs or ZnO bulk, are shown in Fig. 4E and F. In drought-stressed seedlings, soluble carbohydrate concentrations in shoots decreased significantly to 32.4% and 77.4% at 80% and 60% FC, respectively. In contrast, the soluble carbohydrate content of the roots significantly increased to 116.5% at 80% FC compared with that of the control seedlings. Seed priming with ZnO NPs under 80% FC stress increased soluble carbohydrate concentrations in the shoot to 197.7%, whereas root concentrations were suppressed to 49.6%. Additionally, seed priming with bulk ZnO under severe drought stress at 60% FC reduced soluble carbohydrates to 57% in shoots and 51% in roots at 80% FC, whereas severe drought stress at 60% FC increased accumulation to 183.4% compared with unstressed seedlings without priming.

### Principal component analysis, hierarchical clustering pattern and correlation analysis

**Fig. 5:**
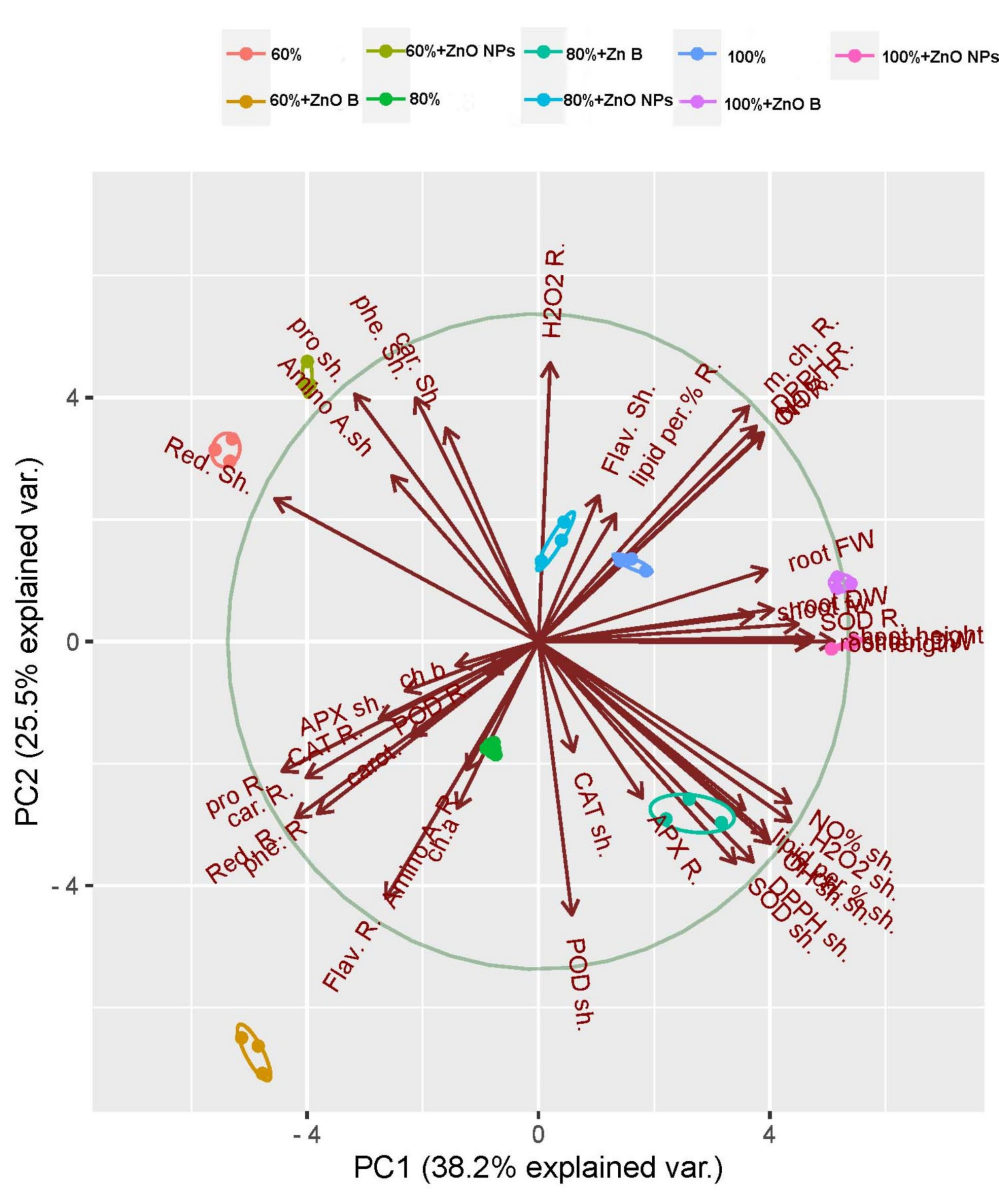
Principal component analysis (PCA) of the studied parameters in shoot and root of wheat seedlings with priming agents (ZnO NPs 60 mg L^− 1^ or ZnO bulk 60 mg L^− 1^) under different levels of drought (100%, 80%, and 60% of field capacity). Fw and Dw, Fresh and Dry weight; Chl a, Chlorophyll a; Chl b, Chlorophyll b; APX, Ascorbate peroxidase, Carot, Carotenoids; CAT, Catalase; POD, Peroxidase; SOD, Superoxide dismutase; Ph, Free phenolic; Flav, total flavonoids; LOX, Lipoxygenase; Lipid per%, Inhibition of lipid peroxide formation %; H_2_O_2_, hydrogen peroxide; Red, Reducing power; DPPH, Total antioxidant activity; OH, Hydroxyl radical (OH.^−^) scavenging %; NO%, Nitric oxide scavenging %; Car, Soluble carbohydrates; Pro, Soluble protein; Amino A, free amino acids; m. ch, Metal chelating.

Principal component analysis (PCA) was conducted on the experimental dataset, which included 43 physiological variables and 9 treatments, to increase the discrimination power to group the measured traits on the basis of the relationships among treatments (with or without) ZnO NPs or bulk priming amendments under drought stress conditions. Since the first two PCs showed the highest percentage of variance, they were used to create a PCA-based biplot (Fig. 5). Subjection of all the original data of measured traits to PCA provides clear details for all possible correlations (positive & negative) among all assessed traits. The level of trend similarity is shown by the distances between the qualities on both axes. The PCA biplot shows the relationships between all antioxidant abilities and growth indicators (the right-hand half of the biplot in Fig. 5) and soluble metabolites (carbohydrates, proteins, and amino acids), chlorophyll pigments, phenolics and reducing power (the left-hand half). Axis 1 in the PCA biplot accounted for approximately 38.2% of the accumulated percentage, whereas the second axis accounted for approximately 25.5%. The right-hand half of Fig. 5 was strongly affected by the following treatments: 100% FC, 100% FC + ZnO bulk priming and 80% FC with ZnO bulk priming or nano priming. Moreover, the left-hand half was strongly affected by 60% FC without priming and 60% FC with ZnO bulk priming or nanoprim priming.

**Fig. 6:**
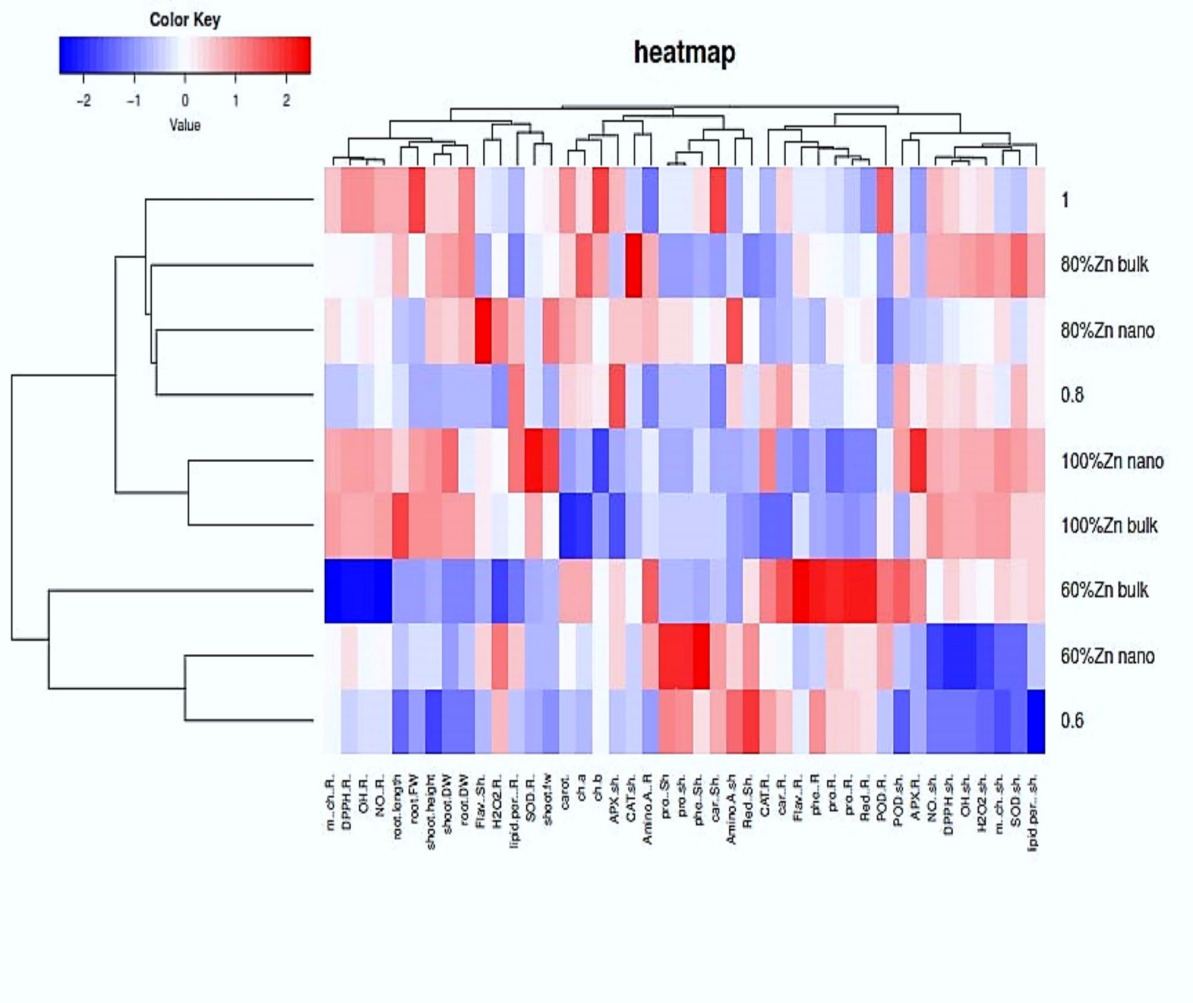
Heatmap showing the saturation of colours indicating effects of the priming agents (ZnO NPs 60 mg L^− 1^ or ZnO bulk 60 mg L^− 1^) under different levels of drought (100%, 80%, and 60% of field capacity) on the studied parameters in shoot and root of wheat seedlings. Fw and Dw, Fresh and Dry weight; Chl a, Chlorophyll a; Chl b, Chlorophyll b; APX, Ascorbate peroxidase, Carot, Carotenoids; CAT, Catalase; POD, Peroxidase; SOD, Superoxide dismutase; Ph, Free phenolic; Flav, total flavonoids; LOX, Lipoxygenase; Lipid per%, Inhibition of lipid peroxide formation %; H_2_O_2_, hydrogen peroxide; Red, Reducing power; DPPH, Total antioxidant activity; OH, Hydroxyl radical (OH.^−^) scavenging %; NO%, Nitric oxide scavenging %; Car, Soluble carbohydrates; Pro, Soluble protein; Amino A, free amino acids; m. ch, Metal chelating.

The two-sided dendrogram obtained from the cluster analysis revealed that all the investigated treatments and measured traits were grouped into different subclusters (Fig. 6). The visual plot of the heatmap is used to find complex associations among multiple parameters under different treatments. It is very useful to add hierarchical clustering to the heatmap as an attempt to arrange items in a hierarchy according to the similarity among them. Hierarchical clustering analysis and heatmaps revealed significant differences among the treatments on the left side and the parameters on the top (Fig. 6). Compared with drought treatment without priming, the application of the priming agents ZnO bulk or ZnO NPs changed the response of all the studied growth, physiological and yield attributes under different drought conditions (Fig. 6). The growth attributes clustered with root antioxidant enzymes (SOD), root antioxidant abilities (metal chelation, NO scavenging, H_2_O_2_ scavenging, and lipid peroxidation inhibition) and total antioxidants (DPPH), as observed in the heatmap.

**Fig. 7:**
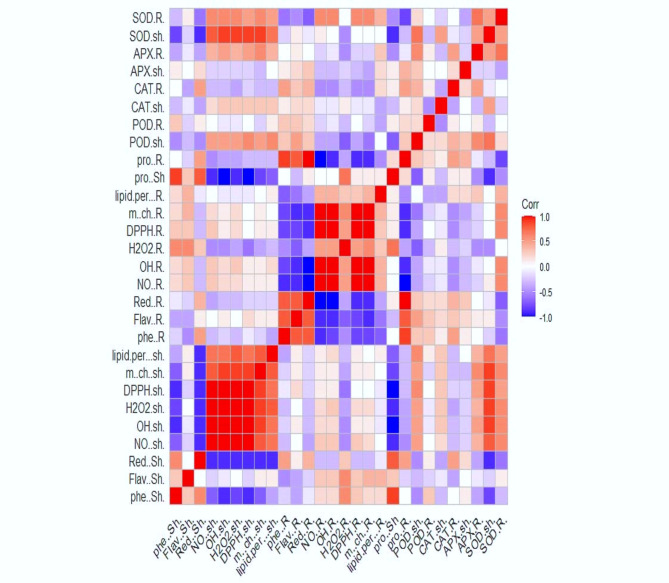
Correlation matrix of the 28 measured traits of the studied parameters in shoot and root of wheat plants with priming agents (ZnO NPs 60 mg L^− 1^ or ZnO bulk 60 mg L^− 1^) under different levels of drought (100%, 80%, and 60% of field capacity). The increasing colour intensities illustrate a higher correlation coefficient. Fw and Dw, Fresh and Dry weight; Chl a, Chlorophyll a; Chl b, Chlorophyll b; APX, Ascorbate peroxidase, Carot, Carotenoids; CAT, Catalase; POD, Peroxidase; SOD, Superoxide dismutase; Ph, Free phenolic; Flav, total flavonoids; LOX, Lipoxygenase; Lipid per%, Inhibition of lipid peroxide formation %; H_2_O_2_, hydrogen peroxide; Red, Reducing power; DPPH, Total antioxidant activity; OH, Hydroxyl radical (OH.^−^) scavenging %; NO%, Nitric oxide scavenging %; Car, Soluble carbohydrates; Pro, Soluble protein; Amino A, free amino acids; m. ch, Metal chelating.

A visual plot of the correlation analysis was used to visualize positive and negative correlations among multiple parameters under different treatments (Fig. 7). Strong negative correlations were observed between proteins and reducing power in shoots from one side and shoot antioxidant abilities (metal chelating %, NO scavenging %, H_2_O_2_ scavenging %, OH scavenging %, lipid peroxidation inhibition %, and total antioxidants DPPH). Strong positive correlations were detected among all these parameters (metal chelation, NO scavenging, H_2_O_2_ scavenging, OH scavenging, lipid peroxidation inhibition, and total antioxidant (DPPH)) and SOD in the shoots and roots.

## Discussion

This study demonstrated how Zn seed priming, which involves the application of either bulk ZnO or ZnO NPs, mitigates the negative effects of drought on wheat seedlings. To investigate the mechanisms of Zn-mediated drought resistance, we assessed various growth, physiological, and biochemical parameters essential for wheat survival under water deficit conditions. Under natural and agricultural conditions, plants face different environmental challenges during their growth and development. Among these stresses, drought is the most damaging environmental stress to plant productivity. Water makes up between 80 and 95% of the fresh biomass in a plant’s body and is essential for various physiological functions, including many aspects of plant growth, development, and metabolism^59,60^.

The absorption band at 3438 cm^− 1^ can be assigned to the stretching and bending vibrations of OH groups of absorbed water molecules during the measurements^61,62^. The other absorption band at 1643 cm^− 1^ in the FTIR spectrum of our samples is also correlated with ZnO nanoparticles^63^. The band at 447 cm^− 1^ is assigned to the stretching vibration of Zn-O in the octahedral coordination, which also confirms the wurzite hexagonal structure formation of the ZnO nanopowder^64^. The gaseous phases of an isolated molecule, ZnO HNB/Iso, are described by the equation $\:{\lambda\:}_{Cal}.=\:0.019{\lambda\:}_{Exp}.+13.25$; the correlation coefficient (R2 = 0.008) indicates the direct correlation between the calculated $\:{(\lambda\:}_{Cal}.)\:$and experimental wavenumbers ($\:{\lambda\:}_{Exp}.$)^65^.

All the diffraction peaks are indexed to the cubic crystalline phase of the ZnO NPs, space group F23 (196)^66^. Among these planes, the (202) surface, with the highest intensity, presents the least surface energy and is thermodynamically and electrostatically the most stable^67^. The calculated values as well as the observed d-space values are in good agreement with the reported 96-412-4787 card. Chen et al.^68,]^ Singh et al.^69^ reported very similar results for ZnO NPs prepared *via* a ball milling process using commercial ZnO purchased from Chem Lab NV Company, Belgium (99.5% purity, 81.37 g/mol and a density of 5.61 g/cm^3^). The Microsoft applications TDDFT-DFT and Crystal Sleuth were utilized to indicate peak lines that were estimated through diffraction and were close to the measured results^70^. The mean crystallite size ($\:{D}_{Av}$) of the powder sample was estimated via Scherer’s formula $\:{D}_{Av}=0.9\lambda\:/\left(\beta\:\:cos\:\theta\:\right)$^71^, where λ is the X-ray wavelength (0.154 nm), β is the peak width at half maximum (in radians) and θ is the angle of the corresponding peak.

The experimental and measured PXRD patterns of the zinc oxide nanoparticles were compared in terms of their corresponding experimental X-ray structures^72^. Although experimental and PXRD models differ slightly in the locations and intensities of specific peaks, this article focuses primarily on their fundamental similarities. Therefore, when comparing experimental and measured data, only the essential attributes should be taken into account^36^. The experimental PXRD pattern can be influenced by various factors associated with the microstructural properties of the particle sample, apart from the instrumentation and data collection procedure. The experimental and computed PXRD patterns of the zinc oxide nanoparticles are in good agreement, as determined by a comprehensive assessment. This confirms the accuracy of the PXRD patterns of the fabricated material^73^.

According to the results of the present study, increasing the level of drought caused drastic impacts on the growth parameters of both the shoots and roots of wheat seedlings when seed priming with ZnO NPs or ZnO bulk increased these traits under water stress conditions. Similar findings were reported by Miranshahi and Sayyari^74,]^ who reported that drought stress reduced all growth indices of summer savory (*Satureja hortensis*) plants. Under conditions of water deficiency, the decrease in cell expansion may have contributed to the decline in plant height and dry weight. Additionally, low-water conditions trigger a decrease in plant cytokinin levels, which inhibits shoot growth and promotes root development. As reported by Agurla et al.^75,]^ when rice plant cells are exposed to water shortage conditions, stomatal closure results from an increase in ABA levels. An increased root-to-shoot ratio in drought-stressed rice plants is correlated with lower cytokinin levels.

Sadeghzadeh^76^ reported that zinc (Zn) is a crucial micronutrient essential for plant functions such as growth, hormone production, and internode elongation; consequently, adequate levels of this element are undoubtedly necessary for normal development. In addition, the levels of gibberellin and auxin, which stimulate growth, are increased in seeds after nanoparticle priming^77^. In general, the involvement of nanoparticles could be responsible for the improved growth characteristics observed in maize plants, such as increased plant height and number of leaves. These nanoparticles are essential because they suppress the ethylene signaling pathways of plants. Moreover, they help increase the level of gibberellic acid, which promotes cell elongation, eventually resulting in improved maize plant growth^78^.

According to the findings of this study, various levels of drought (80% and 60% FC) significantly reduced photosynthetic pigment concentrations in stressed seedlings, especially at high level (60% FC), compared with those in the control group. However, seed priming with either ZnO NPs or bulk ZnO stimulated pigment concentrations in the leaves of stressed plants. This finding is consistent with that of Jorge et al.^79,]^ who suggested that complete stomatal closure caused by severe drought severely reduces photosynthesis while only moderately inhibiting or even promoting the respiration rate. Indirectly, drought stress decreases CO_2_ intake through stomata, which rapidly close when plants experience water stress and directly modify their biochemical reactions. Consequently, drought stress restricts photosynthesis and limits plant vegetative development by restricting the movement of photosynthetic components^80^. A study conducted by Venkatachalam et al.^81^ revealed that, compared with the control, treatment with ZnO nanoparticles significantly increased the concentrations of photosynthetic pigments (Chl *a*, *b*, and carotenoids) in cotton plants. Compared with the control, the application of ZnO nanoparticles to cotton plants increased the concentrations of photosynthetic pigments, such as carotenoids and chlorophyll. Although zinc is a cofactor of enzymes involved in chlorophyll biosynthesis, it promotes chlorophyll. In particular, regardless of the water conditions, various forms of zinc (ionic, nano, and bulk oxides) can promote the formation of chlorophyll in crop plants^82,83^. Similarly, Wang et al.^84,85^ reported that, after exposure to ZnO N/B particles, certain genes related to photosynthesis, the generation of chlorophyll, and the photosystem structure increased at low concentrations and decreased at relatively high concentrations. This could be attributed to the role of zinc in plant cell division and the integrity of the leaf membrane^86^.

By bursting reactive oxygen species (ROS), plants can effectively defend themselves against stress in the early stages of adverse environmental conditions. ROS subsequently behave as signaling molecules that activate the defense mechanisms of the organism^87,88^. When the ROS concentration exceeds the threshold, however, the plant loses its ability to react to osmotic stress; in severe cases, this may result in wilting or even death^89,90^. The main components of the enzymatic scavenging system are ascorbate peroxidase (APX), peroxidase (POD), catalase (CAT), and superoxide dismutase (SOD)^91^.

In the present study, drought stress increased the activities of APX, CAT, and SOD in the leaves, but the same conditions decreased the POD and SOD activities in the roots of the wheat seedlings compared with those in the control seedlings. However, priming with bulk ZnO or NPs significantly reduced the activities of APX, CAT, and POD in leaves and roots at 80% FC but increased their activities at 60% FC compared with those of nonprimed stressed plants. These results can be explained by the conclusions drawn from the study of Sistu et al.^92,]^ which revealed that, under drought stress, tolerant genotypes presented increased antioxidant enzyme activity in comparison with that under normal conditions. Research has shown a strong correlation between elevated antioxidant enzyme activity and chickpea drought tolerance^93^.

These results agreed with those of García-Gómez et al.^94,]^ who reported that APX and POD activities in pea leaves increased while CAT activity decreased in response to exposure to ZnO NPs. Furthermore, Adrees et al.^95^ reported that by lowering ROS activity and preserving physiological processes in crop plants, nanoparticles can mitigate the negative effects of drought stress. The role of these genes in signaling pathways and defense mechanisms under stressful circumstances has been extensively demonstrated in earlier research. Manvelian et al.^96^ reported that drought stress increased POX activity in all safflower cultivars, interacting with zinc application and affecting POX activity. Moreover, the capacity of zinc to protect membrane lipids and proteins from free radicals and other byproducts of intracellular reduction events may be the reason for this increase in membrane lipids and proteins from free radicals, and other byproducts of intracellular reduction events may account for this increase in membrane integrity.

As alternatives, phenolic compounds, a nonantioxidant system, can be produced in various plant parts to control water status and maintain ROS generation below harmful levels during both abiotic and biotic stresses. Under drought conditions, the production of these metabolites can increase or decrease depending on the severity of stress and a plant’s ability to begin these physiological processes^97^. According to a previous study Ghani et al.^80^, ZnO NPs increase the activity of antioxidant defense mechanisms, which are vital for scavenging these harmful radicals. These systems include both enzymatic (CAT, POD, PAL, DHAR, MDHAR, GR, etc.) and nonenzymatic (AsA and GSH) systems. In addition, Hasanuzzaman et al.^98^, and Ahanger et al.^99^, the stability of O^− 2^ and H_2_O_2_ prevents the generation of hazardous redials (OH^−)^, which stabilize cellular and membrane functions.

A recent experiment revealed that, compared with control conditions, severe drought conditions increased the levels of free phenolics in the shoots and roots while simultaneously reducing total flavonoid accumulation in the shoots. Furthermore, seed priming with ZnO NPs and bulk ZnO resulted in higher concentrations of free phenolics in the shoots and roots of stressed plants. These results align with the findings of Hossain et al.^100,]^ who reported that, under abiotic stress, polyphenols, particularly total phenolics and flavonoids, are important for scavenging reactive oxygen species (ROS) and can serve as good sources of antioxidants, which are crucial for human health, while simultaneously increasing the nutritional content of seeds. In contrast, Alvarenga et al.^101^ demonstrated that severe water stress results in a significant reduction in the total flavonoid content of pepper rosemary (*Lippia sidoides* Cham.) leaves after 60 days of cultivation. Previous experiments have shown that plants treated with ZnO NPs or bulk Zn presented increased concentrations of flavonoids and phenols, as well as increased activity of some antioxidant enzymes. Some scientists have suggested that Zn plays a role in stimulating the expression of genes associated with the synthesis of antioxidants and secondary metabolism^102,103^.

The scavenging properties of plant extracts have been widely tested *via* the use of DPPH, a stable nitrogen-centered free radical^104^. The results of a recent study revealed that a severe level of drought (60%) had significant suppressive effects on the percentage of the total antioxidant content (DPPH) of shoots and roots in drought-stressed plants compared with that in the controls; moreover, seed priming with ZnO NPs under stressed conditions significantly diminished their accumulation in shoots and had positive effects on their percentage in the roots. Conversely, the priming of seeds with bulk ZnO under stressed conditions led to a slightly significant increase in its concentration in the shoots and roots. On the other hand, the obtained results are consistent with the findings of Król et al.^105,]^ who reported that grapevine seedlings under drought stress presented lower levels of reduction in the levels of antioxidant substances in their tissues. These findings are in agreement with those of Baskar et al.^106^, who reported that the free radical scavenging activity of *Abelmoschus esculentus* treated with ZnO NPs remained unchanged up to 250 mg/L. As the NP concentration increased, the values of radical scavenging decreased in that order^106^. Furthermore, the electrostatic interaction between negatively charged bioactive substances (COO^−^, O^−^) and positively charged NPs (ZnO = Zn^2+^ + O_2_) found in plant extracts is likely the cause of the antioxidant efficiency of ZnO NPs against DPPH^107^. Numerous researchers have reported that foliar application of Zn upregulates Zn finger protein expression, resulting in increased antioxidant activity and reduced ROS generation^108^.

Koobaz et al.^109^ elucidated the hydroxyl radical scavenging abilities of various naturally occurring vacuolar substances. According to Michelet et al.^110^, hydrogen peroxide is the most stable reactive oxygen species (ROS) that rapidly oxidizes other substances. The Haber‒Weiss reaction is accelerated by the high level of H_2_O_2_ in plant cells, which also influences the generation of hydroxyl radicals, leading to lipid peroxidation^111^. The results of this study indicated that, compared with the control seedlings, drought stress significantly inhibited the scavenging of hydroxyl radicals (OH. %) and hydrogen peroxide (H_2_O_2_) in the shoots of stressed seedlings. Under stressful conditions, compared with nonprimed plants, seedlings primed with ZnO NPs or bulk ZnO had lower percentages of scavenged material in the shoots and roots.

Generally, antioxidant activity is derived from an antioxidant composition, such as the number of hydroxyl groups, which play an essential role in the capacity of antioxidants to eliminate free radicals. The action of ZnO NPs on radicles can be described in two ways: (1) the function of zinc in metabolic processes and (2) the role of reactive oxygen species (ROS)^112^. Recent findings are consistent with those of Behtash et al.^113^, who postulated that the negative correlations between these damaging substances and the increased activity of antioxidant enzymes support the theory that the application of zinc may help cells detoxify by breaking down free radicals such as H_2_O_2_. Zn stabilizes biological membranes and can alter their permeability when it reacts with the sulfhydryl groups of membranes.

A study by Nabi et al.^114^ revealed that nitric oxide (NO) is considered an essential signaling molecule that triggers numerous physiological and developmental processes in plants, from photosynthesis and redox equilibrium to germination and senescence. In addition, NO plays a vital role as a functional signaling molecule in plants, and several studies have demonstrated that NO has a protective effect in response to abiotic stress and is directly linked to the reduction of reactive oxygen species (ROS) in plants^115,116^. Recent findings have shown that drought-stressed plants exhibit significant inhibition of NO scavenging in both shoots and roots. Seed priming with ZnO NPs under drought-stressed conditions was more effective at increasing the percentage of NO scavenging in shoots than in roots. However, negative effects on the percentage of NO scavenging were observed in the roots of plants grown under drought stress in combination with seed priming with bulk ZnO. The severity of drought stress, duration of exposure, and plant growth stages all influence the generation of NO. For example, compared with well-irrigated plants, *Cucumis sativus* presented a 75-fold increase in NO production during the first 10 h of drought exposure, which further increased to 190-fold after 17 h^117^. Zhao et al.^118^ clearly and extensively demonstrated that important enzymes involved in carboxylic acid, amino acid, and glucose metabolism were significantly downregulated when the intercellular NO concentration decreased under drought stress (PEG + c-PTIO). Previous research has shown that ZnO NPs increase NO levels in *Brassica juncea* but have no significant effect on *Brassica napus* roots, whereas Zn^+ 2^ (in the form of ZnSO_4_) treatment increases NO levels in Arabidopsis seedlings^119,120^. This may be explained by some studies indicating that elevated zinc levels cause NO to accumulate, which can increase the activation of many defense systems. Accordingly, NO seems to function as a signal that aids in Zn-mediated reactions, enabling plants to withstand elevated levels of this metal^121^. An enhanced antioxidant response is generally believed to be closely associated with NO-mediated protection against Zn toxicity^122^.

Water stress enhances MDA generation, which harms the membrane structure and decreases membrane stability^123^. In plants, lipid peroxidation is often used to indicate oxidative damage^124^. In the present study, under drought stress conditions at all levels, the inhibition of lipid peroxidation decreased more in the shoots than in the roots. In contrast to drought-stressed plants without priming, seeds primed with ZnO NPs under stress conditions presented an increased percentage of lipid peroxidation inhibition and increased shoot and root abilities to protect membranes. On the other hand, the protective role of priming with bulk ZnO was evident in the shoots, whereas in the roots, it decreased to the lowest percentage of lipid peroxidation inhibition. The ability of antioxidants to scavenge free radicals and prevent lipid peroxidation, thus protecting the cell membrane, has been associated with an increase in antioxidant levels. This finding is consistent with the results of Gharibi et al.^125^ on *Achillea millefolium* grown under severe drought stress conditions. Severe stress may increase the synthesis of phenolics, flavonoids, and antioxidants, which in turn suppress the production of MDA and H_2_O_2_ within the cell and accelerate the elimination of free radicals. A hypothesis presented by Foroutan et al.^126^ is that ZnO NPs treatment maintains the integrity of the cell membrane by reducing the amount of MDA present in moringa plants.

Some researchers have reported that drought stress conditions cause the accumulation of high amounts of osmoprotectants, such as proline, soluble carbohydrates, and soluble proteins. These compounds help with osmotic adjustment, maintain cell turgor, and protect various cell structures. This accumulation might significantly improve the ability of cotton and maize plants to withstand drought^127,128^. The present study revealed that drought stress, especially under severe conditions (60% FC), increased the accumulation of soluble protein and amino acids in the shoots and roots. Compared with those of stressed plants without seed priming, the shoots of plants grown under drought stress with seed priming with ZnO NPs presented a similar response, whereas compared with those of stressed plants without seed priming, the accumulation of plants with bulk ZnO under stressed conditions decreased. The roots of those plants grown under the previously mentioned conditions presented significant increases in their concentrations, whether by priming with ZnO NPs or bulk ZnO under stressed conditions. These findings are in agreement with those of a previous study by Ghani et al.^80,]^ which demonstrated that, owing to a reduction in the negative effects of drought stress, zinc oxide nanoparticles increased the protein content of cucumber seedlings. Zn is essential for ionic interchange and enhances nitrogen assimilation and protein content. Zinc also plays a regulatory cofactor role in protein synthesis, which is vital for tryptophan synthesis and important in regulating membrane potential and structure^83^.

In the present study, the levels of soluble sugars were significantly reduced in the shoots and increased in the roots under drought stress. However, when the seeds were primed with ZnO NPs under stressed conditions, the concentrations of soluble sugars in the shoots were positively affected, whereas those in the roots were significantly lower. Conversely, seeds primed with bulk ZnO combined with a severe level of drought presented decreased concentrations of soluble sugars in the shoots, whereas under the previously mentioned conditions, soluble sugars increased their concentrations in the roots compared with stressed seedlings without priming. This finding is consistent with the findings of Dehghan et al.^129,]^ who noted that, as a result of stomatal closure and decreased expression of genes associated with the Kelvin cycle during drought stress, glucose and fructose levels in leaves increase. Dependency reduces the stabilization of carbon during stress according to the diminished synthesis of triose phosphates, and their transfer from the chloroplast is likely the reason for the decreased expression of these genes. On the other hand, the increase in soluble sugars in wheat cultivars treated with ZnO NPs under drought stress could be attributed to various factors, including hindering the conversion of insoluble carbohydrates to soluble sugars, increasing sucrose transport, decreasing the sucrose-to-starch ratio, and inhibiting starch breakdown outside the leaves for osmotic adjustment during water deficit^130,131^.

## Conclusion

In conclusion, oxidative stress and cell damage are caused by drought, which disrupts the redox equilibrium within plant cells. In response to drought stress, plants change their morphology, molecular pathways, biochemistry, and physiology. The findings of this study suggest that priming wheat seeds with ZnO NPs or ZnO bulk could be a viable option for commercialization in agriculture. This study investigated the effects of priming wheat seeds with bulk zinc or ZnO NPs (60 mg/L) on wheat (*Triticum aestivum* L.) plants under different levels of drought stress (100%, 80%, and 60% FC) at the seedling stage. The IR and XRD analysis of zinc oxide nanoparticles is supported by our improved knowledge of the electronic characteristics of nanocomposite substances, which is made possible by the capacity to calculate and analyze the energy gaps *via* TD‒DFT collocations. The results revealed that under drought stress (80% and 60% FC), the growth parameters, antioxidant enzymes, nonenzymatic antioxidants (such as total phenolics and flavenoids), free radical scavenging abilities (%OH, %NO, and %metal chelating), soluble carbohydrates, and photosynthetic pigments of leaves were significantly inhibited in both the shoots and roots. However, the same conditions enhanced other traits in the shoots and roots, such as the catalase enzyme, reducing power, soluble protein, and free amino acids. Priming the seeds with Zn, either as ZnO NPs or ZnO bulk, improved the performance of the wheat plants under drought stress. The application of Zn was found to create effective mechanical and physiological barriers, as confirmed by the analysis of antioxidant enzyme activities, nonenzymatic components, free radical scavenging, and osmoprotectant constituents. Recent studies have tended to use nanotechnology as an alternative to organic fertilizers, which have severe harmful effects on soil pollution and plant production. Scientists are making efforts to use these nanofertilizers to improve the growth of economically important agricultural plants under abiotic and biotic conditions. With respect to the positive and negative implications of nano fertilizers in the soil‒plant system, careful utilization of these fertilizers is needed, considering both their effects on the environment and their ability to improve efficiency in each unit area. We are also looking forward to future studies to conduct more experiments on types of nanoparticles synthesized *via* novel safety techniques.

## Data Availability

All data generated or analyzed during this study and included in this published article and its supplementary information file is not publicly available due to its proprietary nature. Supporting data cannot be made openly available. The data can be obtained from the corresponding author on reasonable request.

## Abbreviations

ZnO NPs: Zinc oxide nanoparticles
FC: Field capacity
ROS: Reactive oxygen species
NO: Nitric oxide
